# CRISPR and Gene Augmentation Rescue Trabecular Meshwork Dysfunction in iPSC Models of Lowe Syndrome

**DOI:** 10.1002/advs.77704

**Published:** 2026-09-09

**Authors:** Siyu Chen, Zhiquan Liu, Wenmin Wang, Qing Wang, Tia J. Kowal, Fan Zhang, Grzegorz Walkiewicz, Faruk Hossen, Herbert M. Lachman, Jinqiong Zhou, Yiting Wang, Yang Sun

**Affiliations:** ^1^ Department of Ophthalmology Stanford University School of Medicine Palo Alto California USA; ^2^ Palo Alto Veterans Administration Palo Alto California USA; ^3^ Department of Genetics Albert Einstein College of Medicine Bronx New York USA

**Keywords:** base editing, CRISPR, DNA augmentation, lowe syndrome, OCRL, trabecular meshwork cells

## Abstract

Lowe syndrome is a rare, currently incurable multisystem disorder that affects the eyes, kidneys, and central nervous system. It is caused by mutations in the *OCRL* gene, which encodes an inositol 5‐phosphatase. The disorder remains incurable, and the pathways underlying the ocular symptoms remain poorly understood, largely due to the lack of appropriate models. In this study, trabecular meshwork cell models of Lowe syndrome were generated to test two distinct gene therapy strategies: a mutation‐agnostic *OCRL* DNA augmentation therapy and a patient‐specific CRISPR‐mediated gene correction strategy. The results showed that AAV2‐OCRL demonstrated the highest transduction efficiency in patient iPSC‐derived trabecular meshwork models (iHTM) among the three AAV‐OCRL vectors evaluated, establishing it as a promising delivery vector. Targeted CRISPR‐based gene therapy restored OCRL enzyme activity and corrected cellular defects in patient iPSC‐derived trabecular meshwork cell models. Furthermore, RNA‐sequencing analysis of these models revealed dysregulation of extracellular matrix organization, cell adhesion, focal adhesion, and cytoskeletal regulatory pathways, suggesting that disruption of interconnected ECM‐adhesion‐cytoskeletal networks may contribute to trabecular meshwork dysfunction in Lowe syndrome‐associated glaucoma. These findings indicate the feasibility of *OCRL* gene augmentation and CRISPR‐based gene editing in patient‐derived ocular models and position AAV2‐OCRL as a leading therapeutic candidate for Lowe syndrome.

## Graphical abstract

By modeling Lowe syndrome using patient‐derived iPSCs, this study establishes a human disease model that faithfully recapitulates OCRL deficiency‐associated ciliary and cytoskeletal defects. The model enables evaluation of both mutation‐agnostic DNA augmentation and CRISPR‐mediated mutation correction strategies, both of which restore OCRL function and rescue disease‐associated cellular phenotypes. These findings provide a powerful platform for therapeutic development for Lowe syndrome.

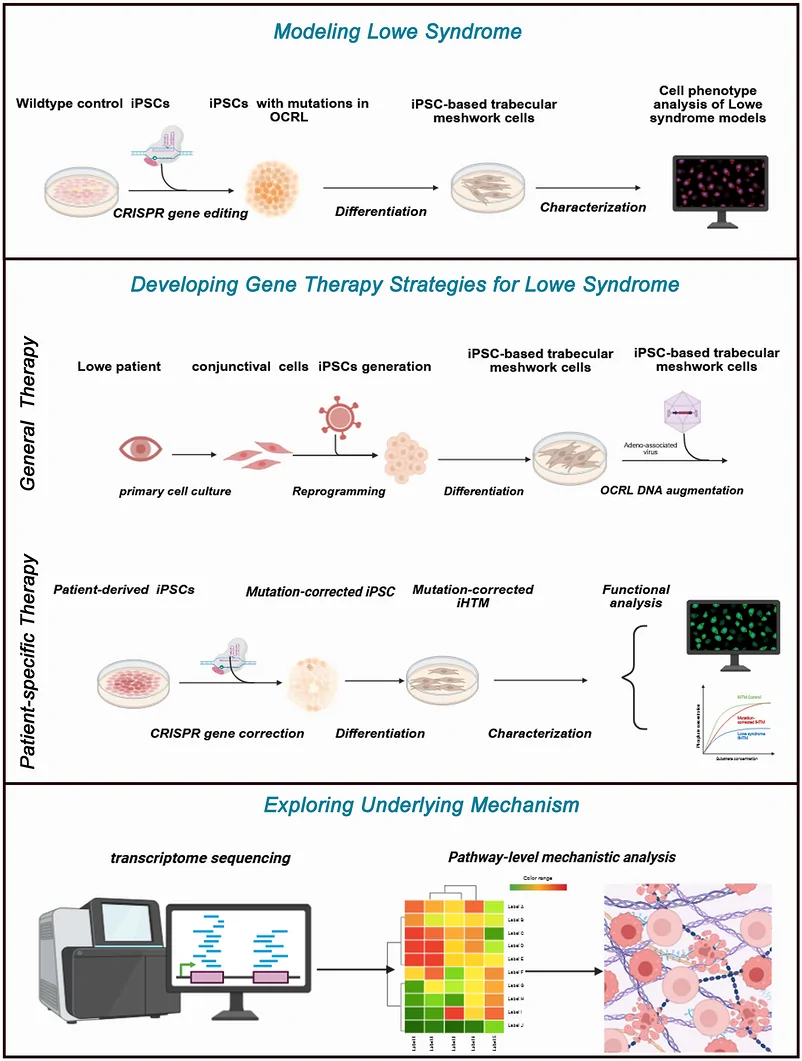

## 1 Introduction

Lowe Syndrome is a rare X‐linked congenital disorder caused by mutations in Oculocerebrorenal syndrome of Lowe (*OCRL*), an inositol 5‐phosphatase that dephosphorylates phosphatidylinositol‐4,5‐bisphosphate (PI(4,5)P_2_) into phosphatidylinositol‐4‐monophosphate. Ocular phenotypes include bilateral dense congenital cataract present at birth and severe glaucoma seen in about 50% of patients [1, 2, 3]. Animal models of mice and zebrafish were made to study the disease [4, 5, 6]. A mouse model of Lowe syndrome was generated by simultaneous disruption of *OCRL* and *Inpp5b*, combined with oocyte injection of a bacterial artificial chromosome (BAC) carrying the human *INPP5B* gene to circumvent embryonic lethality [4]. However, this model fails to recapitulate the full spectrum of Lowe syndrome phenotypes observed in humans. Zebrafish models of Lowe syndrome were established either by retroviral insertion‐mediated disruption of the *OCRL* gene promoter or by transient suppression of OCRL expression using a translation‐blocking morpholino [6, 7]. These models recapitulate several phenotypic features observed in patients with Lowe syndrome, while they are not suitable for evaluating conventional gene therapy approaches, such as DNA augmentation, due to negligible adeno‐associated virus(AAV) transduction efficiency [8]. In addition, several studies have elucidated the mechanisms by which *OCRL* mutations lead to neurological and renal phenotypes [9, 10], the pathways underlying the ocular manifestations, particularly glaucoma, remain largely unexplored due to the lack of appropriate experimental models.

Lowe syndrome is currently incurable, with the management strategies primarily focusing on reducing symptoms and improving the patients’ quality of life. Ocular treatments include cataract removal, prescription of aphakic eyeglasses, and the medical and surgical treatment of glaucoma [3]. In addition, small‐molecule drugs including Rapamycin, statins, and Alpelisib are repurposed to treat Lowe syndrome, while the treatments are not curative. Rapamycin suppresses hyperactive mTOR signaling observed in Lowe syndrome cells. Statins reduce Rho GTPase hyperactivation by limiting prenylation of Rho family proteins, and Alpelisib improves proximal tubule dysfunction in vitro and in a mouse model [11, 12].

DNA augmentation, which delivers a functional copy of the defective genes to restore protein expression, offers a promising approach for genetic diseases by restoring functional causative gene expression and correcting the underlying cellular defects, thus potentially reversing disease phenotypes rather than merely alleviating symptoms. This approach has already shown clinical success in multiple monogenic disorders, including RPE65‐associated inherited retinal diseases, Duchenne muscular dystrophy, and autosomal recessive deafness, demonstrating its feasibility and therapeutic potential [13, 14, 15]. Given the well‐characterized loss‐of‐function mechanism in Lowe syndrome, DNA augmentation offers a rational strategy to restore OCRL function and to prevent disease phenotypes.

CRISPR/Cas has emerged as a revolutionary genome‐editing tool, allowing targeted modification of genes of interest [16, 17, 18]. Adenine base editors (ABE) and prime editors (PE) are two widely used base‐editing tools that combine a catalytically impaired CRISPR‐Cas nuclease with a nucleobase‐modifying enzyme or a reverse transcriptase to achieve precise, programmable base conversions without introducing double‐strand breaks [19, 20]. Given its targeted and precision features, Base editing is suitable for patient‐specific gene therapies for monogenic diseases, with recent success in effectively treating carbamoyl‐phosphate synthetase 1 deficiency [21, 22]. Considering most disease‐causing *OCRL* variants are point mutations, these tools could restore normal gene function in affected cells and correct the underlying cellular defects, making base editing a promising strategy for potentially curative therapy in Lowe syndrome.

To address the lack of patient‐relevant models and therapies for Lowe syndrome, we generated human cellular models by introducing patient‐specific *OCRL* mutations into healthy iPSCs using base editors and prime editors, followed by differentiation into human trabecular meshwork‐like (HTM) cells. In parallel, iPSCs derived from Lowe patient fibroblasts and their TM derivatives were used to evaluate CRISPR‐mediated correction and DNA augmentation strategies. Together, these models enable mechanistic studies of OCRL deficiency and provide a translational platform to assess the efficacy of targeted gene therapies for ocular manifestations of Lowe syndrome.

## 2 Results

### 2.1 Base Editor and Prime Editor‐Mediated Generation of iPSC‐Derived Human Trabecular Meshwork Cell (iHTM) Models of Lowe Syndrome

**FIGURE 1:**
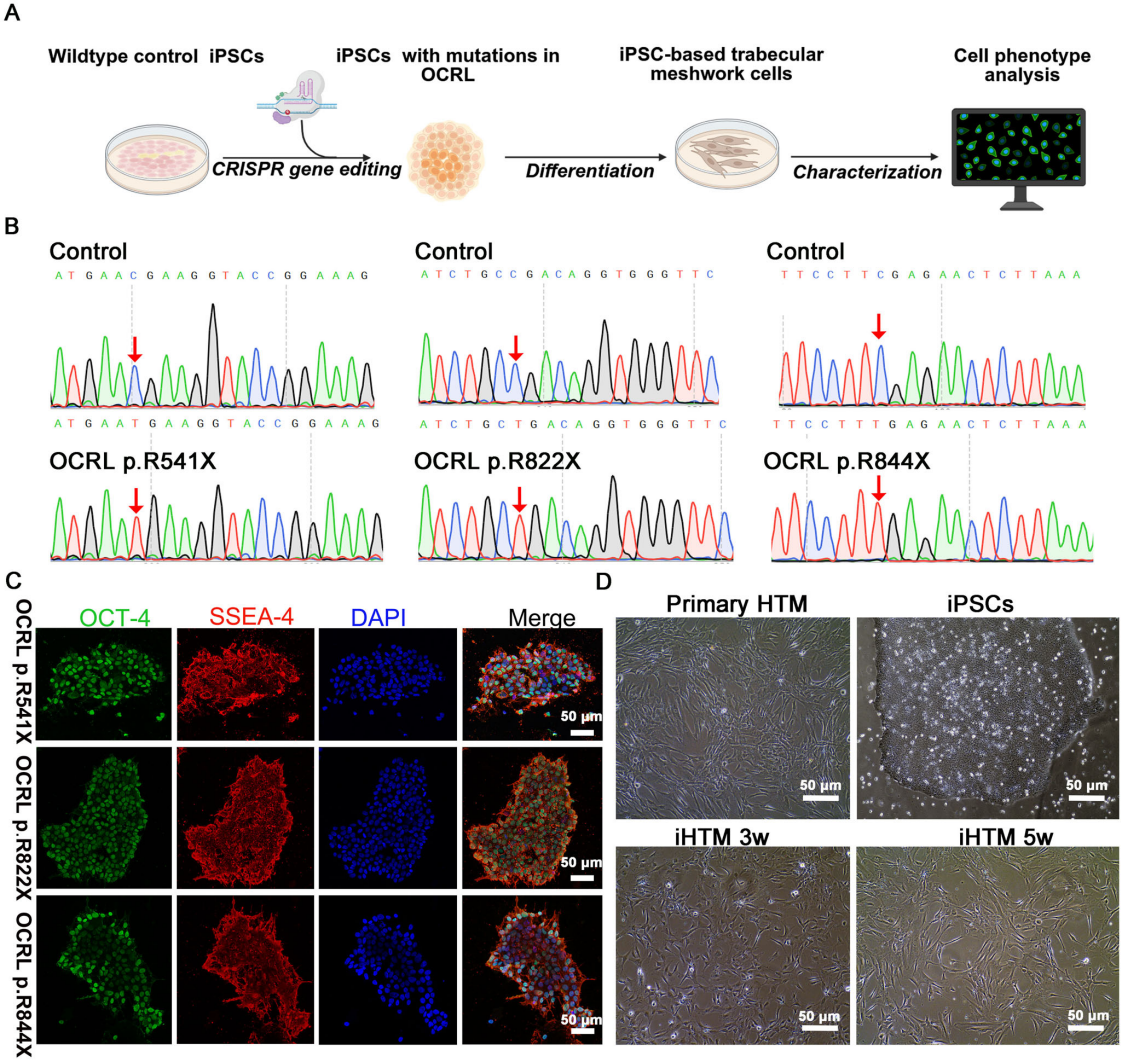
Generation of *OCRL* mutant iPSC to model Lowe Syndrome. (A) Schematic illustration of Lowe syndrome modeling using CRISPR‐based gene editing and subsequent differentiation of induced pluripotent stem cells (iPSCs) into induced human trabecular meshwork (iHTM) cells (created by BioRender). (B) Representative Sanger sequencing chromatograms of iPSC lines harboring Lowe syndrome‐causing mutations in *OCRL*. Red arrows indicate nucleotides before and after CRISPR base editing. (C) Representative immunofluorescence images showing expression of pluripotency markers OCT4 and SSEA4 in iPSCs; nuclei are counterstained with DAPI. Scale bar: 50 µm. (D) Representative phase‐contrast images of primary HTM cells, iPSCs, and 3‐week and 5‐week iPSC‐derived HTM cells (iHTM). Scale bar, 50 µm.

The trabecular meshwork (TM) network regulates aqueous humor outflow and maintains intraocular pressure [23]. Its dysfunction increases outflow resistance, leading to elevated intraocular pressure, which causes retinal ganglion cell degeneration and progressive blindness [24]. Thus, TM cells serve as an appropriate model to study Lowe syndrome‐associated glaucoma. To establish this disease‐relevant model of Lowe syndrome, we first generated isogenic human iPSC lines harboring disease‐causing *OCRL* mutations by introducing Lowe‐causing mutations into a healthy iPSC line using cytosine base editing (CBE) and prime editing (PE) (Figure 1A). Three frequently reported pathogenic *OCRL* mutations (p.R541X, p.R822X, and p.R844X) were selected for modeling Lowe syndrome [25, 26, 27]. Targeted C‐to‐T conversion using a CBE system was employed to generate the OCRL p.R541X mutation, as the target region had only a single cytosine within the editing window, resulting in an editing efficiency of up to 66% (Figure S1A,B). In contrast, PE was used to generate the OCRL p.R822X and OCRL p.R844X mutations because multiple cytosines within the editing window could lead to unintended bystander edits with cytosine base editing. Editing efficiencies of up to 24% and 33% were achieved at the p.R822X and p.R844X target sites, respectively (Figure S1A,B). Three homogeneous iPSC colonies were subsequently selected (Figure 1B). The pluripotency of these iPSC cell lines was validated by immunostaining for OCT4, SSEA‐4, NANOG, and TRA‐1‐60. (Figures 1C and S1C). The absence of OCRL protein in these iPSC lines was confirmed by western blot (Figure S1D). To evaluate potential off‐target effects introduced by the sgRNAs used to generate these cell models, we predicted candidate off‐target sites across the human genome. Our analysis revealed no detectable off‐target editing events (Figure S2). These iPSCs were then differentiated into human trabecular meshwork cells to recapitulate Lowe syndrome in vitro (Figures 1D and S1E).

### 2.2 Validation and Characterization of iPSC‐Derived Human Trabecular Meshwork (iHTM) Cellular Models of Lowe Syndrome

**FIGURE 2:**
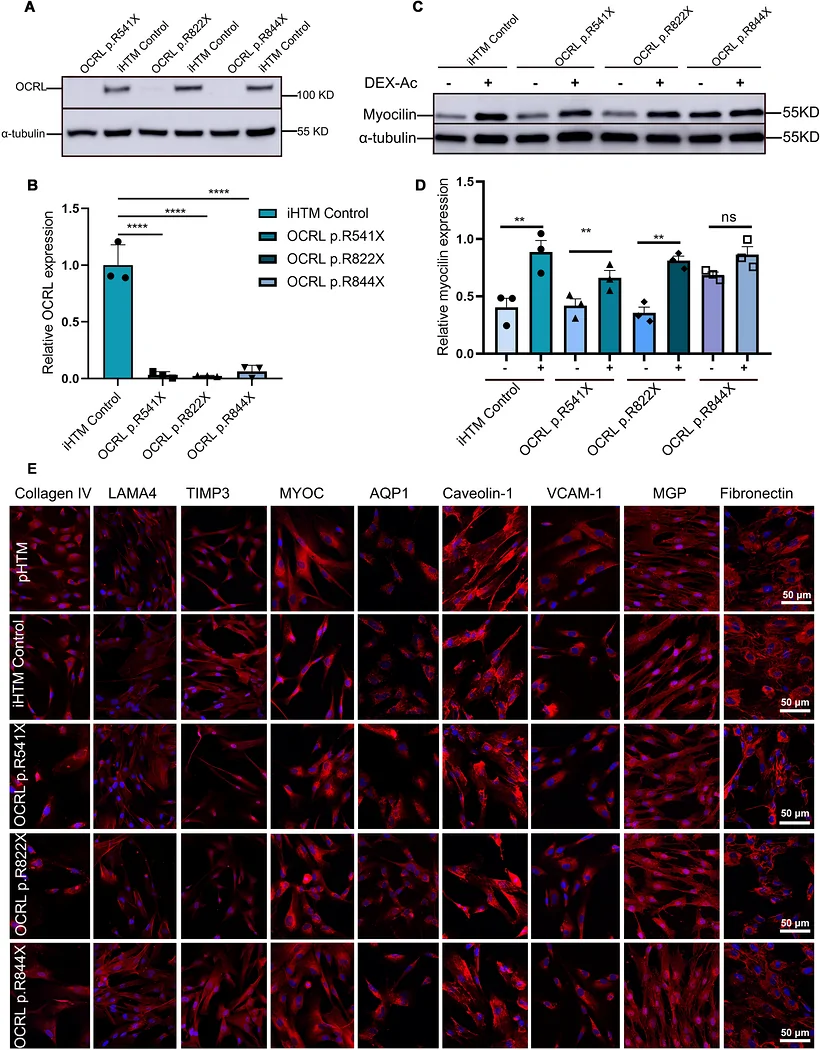
Characterization of Lowe Syndrome iHTM Models. (A, B) Western blot images and quantification showing OCRL protein levels in iHTM cell lines harboring Lowe syndrome‐causing mutations and iHTM Control. (C‐D) Western blot and quantification showing induction of Myocilin expression in iHTM control cells and three distinct *OCRL* mutant cell lines (OCRL p.R541X, OCRL p.R822X, and OCRL p.R844X) following 14 days of incubation in the absence (‐) or presence (+) of 100 nM DEX‐Ac. α‐tubulin was utilized as an internal loading control. Statistical analysis was performed using a two‐tailed paired Student's *t*‐test (^**^ *P* < 0.01; n.s., not significant). (E) Representative immunofluorescence images of trabecular meshwork (TM) markers in primary human trabecular meshwork (pHTM) cells, iHTM control cells, and three *OCRL* nonsense mutant cell lines (p.R541X, p.R822X, and p.R844X). Cells were immunostained for nine previously reported TM biomarkers (red), including Collagen IV, LAMA4, TIMP3, MYOC (Myocilin), AQP1 (Aquaporin‐1), Caveolin‐1, VCAM‐1, MGP (Matrix Gla Protein), and Fibronectin. Nuclei were counterstained with DAPI (blue). Scale bar = 50 µm. Data are shown as mean ± SEM (*n*  =  3 independent experiments). Statistical analysis was performed using one‐way ANOVA followed by Dunnett's multiple‐comparisons test. ^**^, *p* < 0.01; ^***^, *p* < 0.001; ^****^, *p* < 0.0001.

iHTM cell models were generated following a previously published protocol [28]. Western blot analysis confirmed the absence of OCRL protein in the iHTM models of Lowe syndrome (Figure 2A,B). To validate the TM‐like identity and functionality of these iHTM cells, we performed a steroid responsiveness assay, a well‐established functional property of TM cells, which is characterized by increased myocilin (MYOC) expression levels after dexamethasone acetate (DEX‐Ac) treatment [29, 30]. iHTM cells were treated with 100 nM DEX‐Ac for 14 days, followed by analysis of MYOC expression. Consistent with previous studies, DEX‐Ac treatment induced a marked increase in MYOC expression in iHTM cells (Figure 2C,D). Two weeks into differentiation, the iHTM‐like cells expressed trabecular meshwork markers TIMP3, Laminin α4 (LAMA4), and Collagen IV. These cells were smaller than primary human trabecular meshwork cells (pHTM), which reached comparable sizes after five weeks of differentiation (Figure S3A–C). At week 5 of differentiation, iHTM cells were validated by immunostaining for trabecular meshwork markers TIMP3, LAMA4, Collagen IV, MYOC, AQP1 (Aquaporin‐1), Caveolin‐1, VCAM‐1, Matrix Gla Protein (MGP), and Fibronectin, and exhibited morphology comparable to primary human trabecular meshwork cells (pHTM) (Figure 2E).

iHTM cellular models of Lowe syndrome exhibit a disrupted Actin Cytoskeleton Framework and shortened Primary Cilia in iHTM Cells.

**FIGURE 3:**
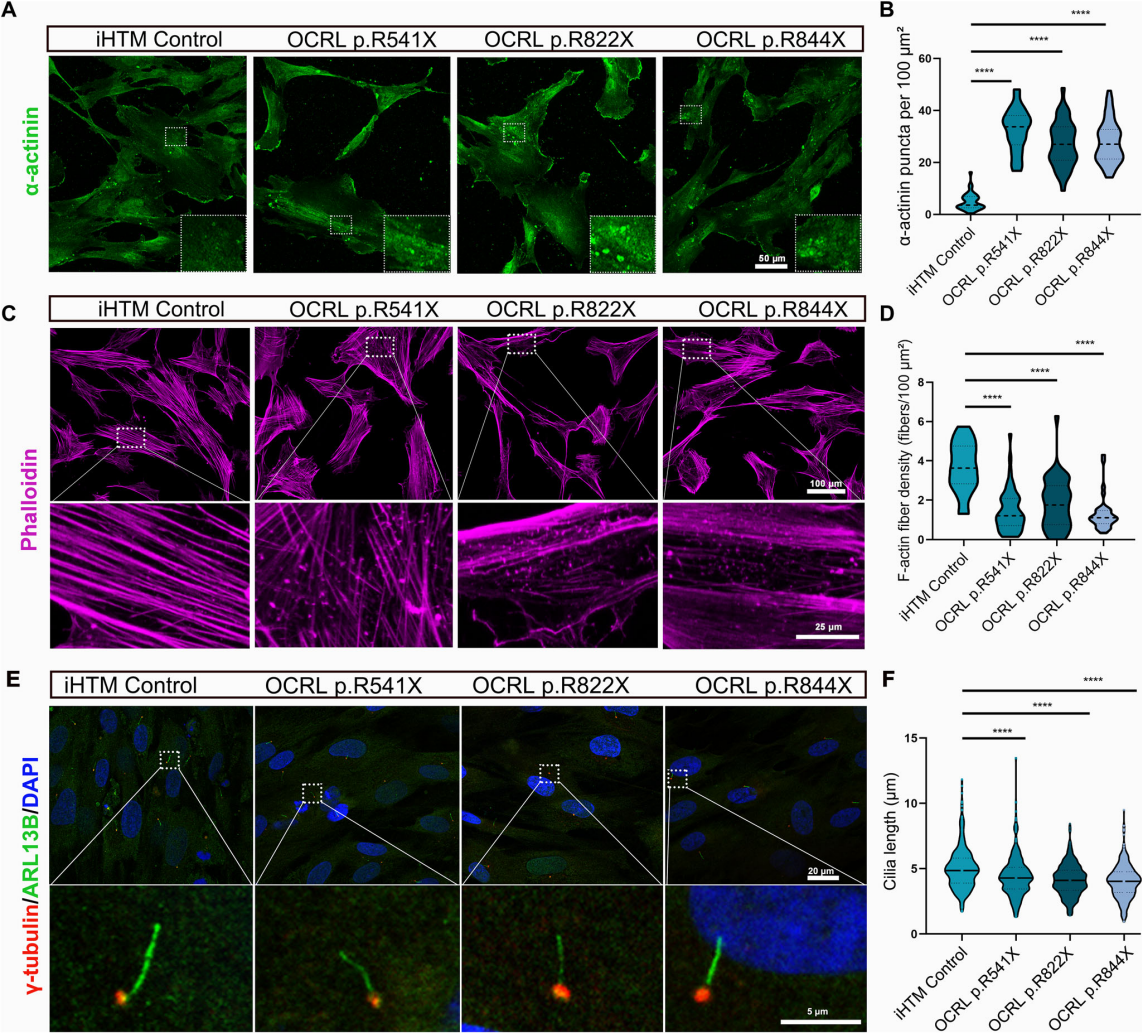
Loss of OCRL disrupts cytoskeletal organization and impairs primary cilia assembly in iHTM cell models of Lowe syndrome. (A) Representative immunofluorescence images of iHTM control and *OCRL* mutant lines (OCRL p.R541X, p.R822X, p.R844X) stained for α‐actinin (green) to evaluate structural organization. Insets show magnified regions of interest detailing normal, smooth α‐actinin alignment in control cells and the abnormal accumulation of α‐actinin puncta in OCRL mutant lines. Scale bar = 50 µm. (B) Quantitative analysis of α‐actinin organization in iHTM Control (n = 36), OCRL p.R541X (n = 29), OCRL p.R822X (n = 40), and OCRL p.R844X (n = 40) lines. α‐Actinin puncta were quantified using Fiji/ImageJ and normalized to cell area, with results expressed as the number of α‐actinin puncta per 100 µm^2^. (C) Representative images and their magnified images showing normal and abnormal F‐actin morphology. Scale bar 100 µm and 25 µm, respectively. (D) Quantitative analysis of F‐actin organization in Control (n = 24), OCRL p.R541X (n = 48), OCRL p.R822X (n = 41), and OCRL p.R844X (n = 34) iHTM cells. F‐actin fibers were quantified using Fiji/ImageJ and normalized to cell area, with results expressed as fiber number per 100 µm^2^. Violin plots represent individual‐cell measurements; dashed lines indicate the median and quartiles. (E) Representative immunofluorescence images of primary cilia in iHTM control cells and three *OCRL* mutant cell lines (p.R541X, p.R822X, and p.R844X). Cells were immunostained for ARL13B (green, ciliary axoneme), γ‐tubulin (red, basal body), and counterstained with DAPI (blue, nuclei). The top row displays wide‐field views, with white dashed boxes indicating the regions selected for higher magnification. Scale bar: 20 µm. The bottom row displays zoomed‐in, high‐magnification insets of individual primary cilia. Scale bars: 5 µm. (F) Quantification of primary cilia length (µm) across iHTM control and OCRL mutant cell lines (p.R541X, p.R822X, and p.R844X). The total number of cilia measured across three independent experiments was n = 383 (iHTM control), n = 321 (p.R541X), n = 368 (p.R822X), and n = 359 (p.R844X). Statistical significance was determined using one‐way ANOVA followed by Dunnett's multiple‐comparisons test, comparing each mutant line against the iHTM control.

It has been well studied that *OCRL* defects disrupt structural and trafficking frameworks, including actin cytoskeleton remodeling and ciliogenesis [31, 32, 33, 34, 35]. We investigated α‐actinin and F‐actin morphology in iHTM models. In wild‐type cells, normal α‐actinin morphology in trabecular meshwork cells is organized in a filamentous pattern along stress fibers; however, in Lowe syndrome patient iPSC‐derived cells, abnormal α‐actinin exhibits disrupted filamentous organization, with irregular α‐actinin puncta accumulation (Figure 3A). Despite cell‐to‐cell variability, quantitative analysis revealed a consistent increase in α‐actinin puncta density across all OCRL‐deficient iHTM cell lines compared with control iHTM cells (32.49, 27.06, and 27.87 vs. 4.90 puncta per 100 µm^2^ for OCRL p.R541X, p.R822X, and p.R844X, respectively) (Figure 3B). Similarly, in wild‐type cells, F‐actin forms a dense network of filamentous structures throughout the cytoplasm; however, in Lowe patient cells, F‐actin exhibits disorganized filaments with fewer or no stress fibers. iHTM cells lacking OCRL showed abnormal cytoskeletal morphology, with disorganized F‐actin networks (Figure 3C), indicating impaired structural integrity of the trabecular meshwork cells. F‐actin fiber density was significantly reduced in all three OCRL‐deficient lines (1.47, 1.83, and 1.29 vs. 3.67 fibers per 100 µm^2^, respectively) (Figure 3D), supporting consistent alterations in actin cytoskeletal organization associated with OCRL deficiency. Furthermore, we investigated primary cilia in OCRL‐deficient and control iHTM cell lines, confirming that cilia length was significantly decreased in the Lowe syndrome cellular models (OCRL p.R541X, mean = 4.42 µm; OCRL p.R822X, mean = 4.13 µm; OCRL p. R844X, mean = 4.01 µm) compared to the iHTM control (mean = 5.05 µm) (Figure 3E,F). Based on these results, we conclude that iHTM models replicate the key OCRL‐related cellular abnormalities characteristic of Lowe syndrome.

### 2.3 AAV‐Mediated *OCRL* Gene Delivery Restores OCRL Expression and Cytoskeletal Integrity and Primary Cilia Length in Patient‐Derived iHTM

**FIGURE 4:**
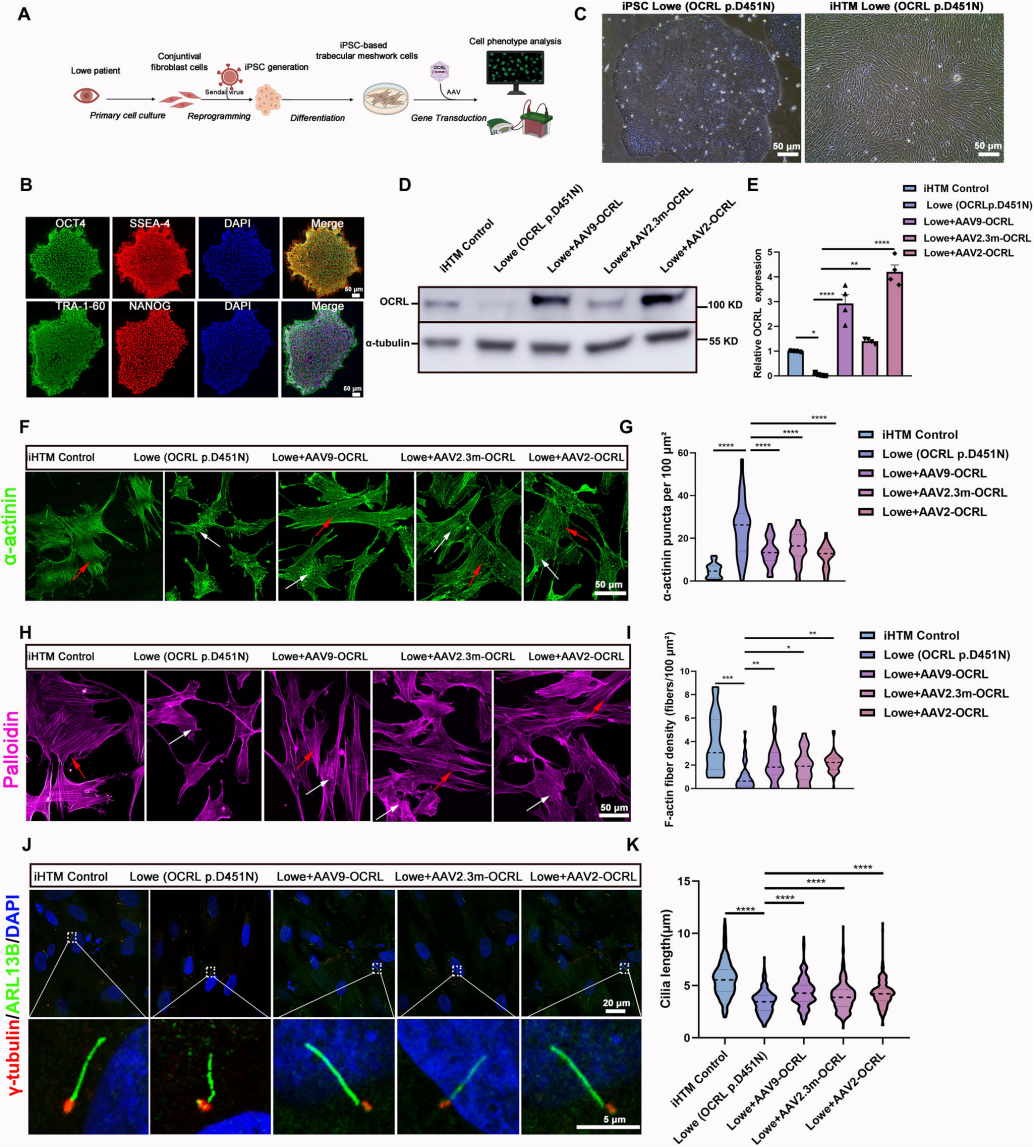
Universal gene therapy approaches in patient iPSC‐derived HTM rescue *OCRL* expression, Cytoskeletal Organization, and primary cilia length. (A) Schematic workflow for generating patient‐specific iPSC‐derived trabecular meshwork‐like cells from conjunctival fibroblasts, followed by DNA augmentation and phenotypic analysis (created by BioRender). (B) Representative immunofluorescence images showing expression of pluripotency markers OCT4, SSEA4, TRA‐1‐60, and NANOG in the patient iPSCs; nuclei are counterstained with DAPI. Scale bar, 50 µm. (C) Representative images show patient‐derived iPSCs before differentiation (up) and the resulting human trabecular meshwork‐like cells (iHTM) five weeks after initiating differentiation (down). Scale bar, 50 µm. (D‐ E) Western blot images and quantification showing OCRL protein levels in patient iHTM Control, Lowe (OCRL p.D451N), Lowe+AAV9‐OCRL, Lowe+AAV2.3m‐OCRL, and Lowe+AAV2‐OCRL. (F) Representative immunofluorescence images showing normal and abnormal α‐actinin morphology in different treatment groups. Red arrows indicate normal α‐actinin morphology; white arrows indicate abnormal α‐actinin puncta accumulation. Scale bar: 50 µm. (G) Quantification of α‐actinin puncta density in control iHTM cells, Lowe syndrome iHTM cells harboring the OCRL p.D451N variant, and Lowe iHTM cells treated with the indicated AAV‐mediated OCRL gene augmentation constructs (n ≥ 27). Dashed lines indicate the median and quartiles. (H) Representative immunofluorescence images showing normal and abnormal F‐actin morphology in different treatment groups. Red arrows indicate normal F‐actin morphology; white arrows indicate abnormal F‐actin morphology. Scale bar: 50 µm. (I) Quantification of F‐actin fiber density in iHTM Control, Lowe (OCRL p.D451N), Lowe+AAV9‐OCRL, Lowe+AAV2.3m‐OCRL and Lowe+AAV2‐OCRL groups (n ≥ 36). Dashed lines indicate the median and quartiles. (J) Representative immunofluorescence images of primary cilia in iHTM control cells, Lowe syndrome mutant cells (OCRL p.D451N), and Lowe syndrome cells treated with various adeno‐associated virus (AAV) vectors expressing wild‐type OCRL (Lowe+AAV9‐OCRL, Lowe+AAV2.3m‐OCRL, and Lowe+AAV2‐OCRL). Cells were immunostained for ARL13B (green, ciliary axoneme), γ‐tubulin (red, basal body), and counterstained with DAPI (blue, nuclei). The top row displays wide‐field views, with white dashed boxes indicating the regions selected for higher magnification. Scale bars: 20 µm. The bottom row displays zoomed‐in, high‐magnification insets of individual primary cilia. Scale bars: 5 µm. (K) Quantification of primary cilia length (µm) across the indicated treatment groups. Dashed and dotted horizontal lines within the violins denote the median and quartiles, respectively. The total number of cilia measured across three independent experiments was n = 237 (iHTM control), n = 213 (p.D451N), n = 218 (Lowe+AAV9 OCRL), n = 276 (Lowe+AAV2.3m OCRL), and n = 230 (Lowe+AAV2 OCRL). Data are shown as mean ± SEM (n = 3). Statistical analysis was performed using one‐way ANOVA followed by Dunnett's multiple‐comparisons test, comparing each mutant line against the Lowe (OCRL p.D451N). ^*^, *p* < 0.05; ^**^, *p* < 0.01; ^***^, *p* < 0.001, n.s, *p* > 0.05.

Given that iPSC‐derived human trabecular meshwork cells provide a reliable model of Lowe syndrome, we used this system to evaluate a gene therapy strategy for OCRL deficiency. Fibroblasts from a one‐year‐old Lowe syndrome patient harboring an OCRL p.D451N mutation were reprogrammed into iPSCs using Sendai virus, differentiated into HTM‐like cells, and subsequently used to assess the efficacy of *OCRL* DNA augmentation approaches (Figure 4A). Reprogrammed iPSC colonies were validated prior to colony picking by live‐cell staining for the pluripotency marker TRA‐1‐60 and the fibroblast marker CD44 (Figure S4A). Pluripotency of the patient‐derived iPSCs was further confirmed by immunostaining for OCT4, SSEA‐4, TRA‐1‐60, and NANOG (Figure 4B). Clearance of the Sendai virus reprogramming vectors was verified after seven passages by RT‐PCR analysis (Figure S4B). Patient‐derived iPSCs were subsequently differentiated into trabecular meshwork‐like cells as described above (Figure 4C). Successful differentiation was confirmed by immunostaining for human trabecular meshwork markers, including collagen IV, TIMP3, and LAMA4, MYOC, AQP1, Caveolin‐1, VCAM‐1, Matrix Gla Protein (MGP), and Fibronectin (Figure S4C). Five‐week post differentiation, the patient‐derived iHTM cells were transduced with three AAV‐OCRL vectors (AAV9‐OCRL, AAV2.3m‐OCRL, and AAV2‐OCRL), each driven by a CMV promoter (Figure S4D). Transduced iHTM cells were harvested for western blot analysis, as well as immunostaining for F‐actin and α‐actinin. AAV‐mediated delivery of the WT *OCRL* gene increased OCRL protein levels in Lowe iHTM cells. Specifically, treatment with AAV9‐OCRL and AAV2‐OCRL restored OCRL expression to levels 2.7‐fold and 4.4‐fold higher, respectively, than in untreated iHTM control cells (Figure 4D,E). In contrast, AAV2.3m‐OCRL treatment resulted in OCRL expression comparable to that in iHTM control cells. Among all vectors tested, AAV2‐OCRL yielded the highest protein expression at the same multiplicity of infection (MOI) of 1 (10^5^ (Figure 4D,E). Similarly, quantitative immunostaining analysis demonstrated that AAV‐mediated *OCRL* expression partially restored actin cytoskeletal organization in Lowe iHTM cells. α‐Actinin puncta density was markedly increased in untreated Lowe (OCRL p.D451N) iHTM cells compared with control iHTM cells (24.63 vs. 4.96 puncta per 100 µm^2^). AAV‐mediated *OCRL* supplementation significantly reduced α‐actinin puncta density to 13.66, 15.89, and 11.49 puncta per 100 µm^2^ following AAV9‐OCRL, AAV2.3m‐OCRL, and AAV2‐OCRL treatment, respectively (Figure 4F,G). Consistently, F‐actin fiber density was substantially reduced in untreated Lowe iHTM cells compared with controls (1.04 vs. 3.85 fibers per 100 µm^2^). AAV9‐OCRL, AAV2.3m‐OCRL, and AAV2‐OCRL significantly increased F‐actin fiber density to 2.24, 1.87, and 2.20 fibers per 100 µm^2^, respectively (Figure 4H,I). Together, these quantitative analyses indicate that AAV‐mediated *OCRL* supplementation partially rescues the altered α‐actinin and F‐actin organization in Lowe iHTM cells. We also investigated primary cilia in Lowe patient iHTM transduced with AAV‐OCRL and control iHTM cell lines (Figure 4J). The results showed that AAV‐OCRL transduction significantly restores primary cilia length in Lowe (OCRL p.D451N, mean = 3.43 µm; Lowe_AAV 9‐OCRL, mean = 4.35 µm; Lowe_AAV2.3m‐OCRL, mean = 3.96 µm); Lowe_AAV2‐OCRL, mean = 4.24 µm) compared to the iHTM control (mean = 5.64 µm) (Figure 4K). Together, these results indicate that restoration of OCRL expression improves cytoskeletal organization and partially restores primary cilia length in Lowe iHTM cells, supporting the therapeutic potential of AAV‐mediated OCRL supplementation.

### 2.4 CRISPR‐Mediated Base Editing Enables Patient‐Specific Correction of *OCRL* Mutations in a Lowe Syndrome Cellular Model

**FIGURE 5:**
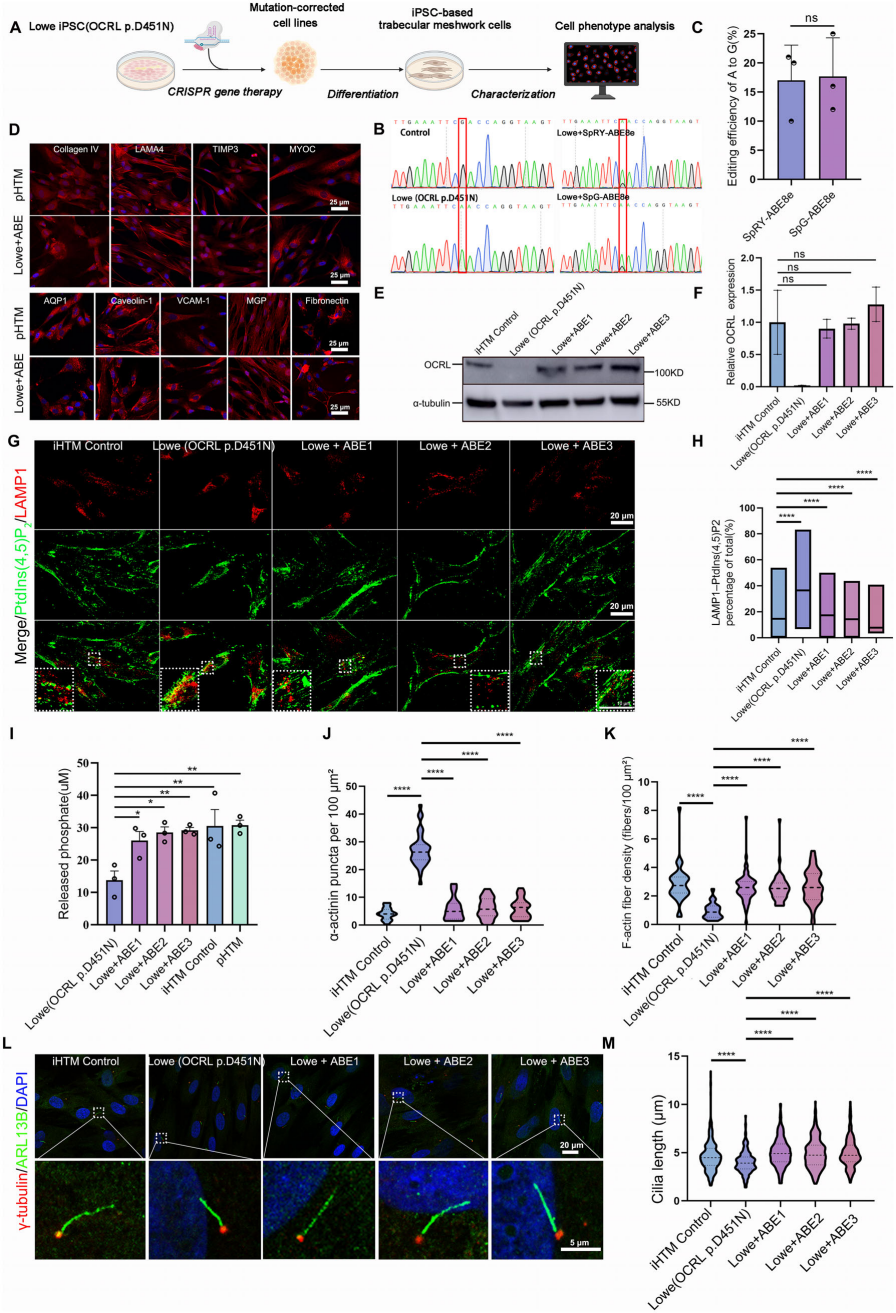
CRISPR base editing‐mediated patient‐specific gene therapy rescues OCRL expression and Cytoskeletal Organization in patient iPSC‐derived HTM. (A) Workflow for CRISPR‐mediated gene correction of Lowe syndrome patient iPSCs and their differentiation into trabecular meshwork cells for phenotypic analysis (created by BioRender). (B) Representative chromatograms show DNA sequences of bulk iPSCs after editing with SpRY‐ABE8e and SpG‐ABE8e editors. Red rectangles highlight the targeted nucleotide positions in each experimental group. (C) Quantification of base editing efficiency. (D) Representative immunofluorescence images showing 5‐week mutation‐corrected iHTMs (Lowe+ABE) expressing nine previously reported TM biomarkers (red), including Collagen IV, LAMA4, TIMP3, MYOC (Myocilin), AQP1 (Aquaporin‐1), Caveolin‐1, VCAM‐1, MGP (Matrix Gla Protein), and Fibronectin. Nuclei were counterstained with DAPI (blue). Scale bar = 25 µm. (E) Representative Western blot images showing OCRL protein levels in three independent CRISPR‐corrected Lowe syndrome iHTM lines (Lowe+ABE1, Lowe+ABE2, Lowe+ABE3) compared to an unaffected iHTM Control. (F) Quantification of OCRL protein levels normalized to α‐tubulin, confirming comparable expression in corrected lines relative to control. (G) Representative confocal immunofluorescence images showing the subcellular distribution and colocalization of OCRL substrate PI(4,5)P_2_ and lysosomal markers in iHTM control cells, Lowe syndrome cells (OCRL p.D451N), and mutation‐corrected Lowe syndrome cells (Lowe + ABE1/2/3). Cells were co‐stained for LAMP1 (red, lysosomes) and PI(4,5)P2 (green). Scale bars = 20 µm. The bottom row displays the merged channels, with white dashed boxes highlighting areas selected for higher‐magnification zoomed‐in insets to emphasize lysosomal phosphoinositide accumulation and rescue. Scale bars = 10 µm. (H) Quantification of LAMP1‐ PtdIns(4,5)P_2_ colocalization percentage (%) across the indicated treatment groups. Data are presented as floating bars showing the minimum‐to‐maximum range, with horizontal lines indicating the median. Statistical significance was determined using one‐way ANOVA followed by Dunnett's multiple‐comparisons test, comparing each cell line against the Lowe (OCRL p.D451N). ^****^ *p* < 0.0001. (I) OCRL enzyme activity in primary HTM, Lowe (OCRL p.D451N), gene‐corrected iHTM cells, and iHTM Control. Data are shown as mean ± SEM (n = 3). (J, K) Quantification of α‐actinin puncta density (n ≥ 21) (J) and F‐actin fiber density (n ≥ 33) (K) in control iHTM cells, uncorrected Lowe syndrome iHTM cells harboring the *OCRL* p.D451N variant, and three independently ABE‐corrected Lowe iHTM clones (ABE1‐ ABE3). Violin plots show the distribution of individual‐cell measurements, with dashed lines indicating the median and quartiles. Statistical analysis was performed using one‐way ANOVA followed by Dunnett's multiple‐comparisons test. ^*^ *p* < 0.05, ^**^ *p* < 0.01, ^***^ *p* < 0.001, ^****^ *p* < 0.0001. (L) Representative immunofluorescence images of primary cilia in iHTM control cells, untreated Lowe syndrome mutant cells (OCRL p.D451N), and mutation‐corrected cell lines (Lowe + ABE1, Lowe + ABE2, and Lowe + ABE3). Cells were immunostained for ARL13B (green, ciliary axoneme), γ‐tubulin (red, basal body), and counterstained with DAPI (blue, nuclei). The top row displays wide‐field views, with white dashed boxes indicating the regions selected for higher magnification. Scale bars: 20 µm. The bottom row displays zoomed‐in, high‐magnification insets of individual primary cilia. Scale bars: 5 µm. (M) Quantification of primary cilia length (µm) across the indicated treatment groups. Dashed and dotted horizontal lines within the violins denote the median and quartiles, respectively. The total number of cilia measured across three independent experiments was n = 355 (iHTM control), n = 386 (p.D451N), n = 231 (Lowe+ABE1), n = 214 (Lowe+ABE2), and n = 221 (Lowe+ABE3). Statistical significance was determined using one‐way ANOVA followed by Dunnett's multiple‐comparisons test, comparing each cell line against the Lowe (OCRL p.D451N). ^****^ *p* < 0.0001.

ABE enables targeted A‐to‐G base conversions at specific genomic loci [16, 18, 20], allowing precise correction of pathogenic point mutations for gene therapy. To develop a patient‐specific gene therapy for the Lowe syndrome patient, gene editing was performed in patient‐derived iPSCs. Mutation‐corrected iPSC clones were subsequently differentiated into trabecular meshwork‐like cells to assess functional rescue (Figure 5A). Sanger sequencing confirmed a G‐to‐A substitution in the patient *OCRL* gene resulting in the p.D451N mutation (Figure 5B). SpRY‐ABE8e and SpG‐ABE8e [16, 36] were used to convert the mutant adenine to guanine, restoring the wild‐type nucleotide. Both editors achieved comparable mutation‐correction efficiencies at the target site, ranging from approximately 10% to 25% (Figure 5C). Mutation‐corrected colonies were subsequently isolated. Screening of the targeted colonies yielded three fully corrected clones alongside a few non‐monoclonal colonies (Figure S5A). The fully corrected clones, along with a healthy control line, were subsequently differentiated into iHTM cells for downstream analysis. iHTM immunostaining confirmed expression of trabecular meshwork markers, including collagen IV, LAMA4, TIMP3, MYOC, AQP1, Caveolin‐1, VCAM‐1, Matrix Gla Protein (MGP), and Fibronectin (Figure 5D). Crucially, these iHTM cell lines exhibited robust steroid responsiveness, mirroring the physiological phenotypes characteristically observed in primary TM cells (Figure S5B,C). Western blot analysis showed restoration of OCR*L* protein expression in base‐edited iHTM cells compared with unedited Lowe iHTM, with α‐tubulin serving as a loading control (Figure 5E). Quantification of OCRL protein levels showed that ABE‐treated groups exhibited increased OCRL expression, reaching levels comparable to iHTM controls (Figure 5F). It has been reported that PI (4,5)P_2_ aberrantly accumulates on the lysosomes when OCRL is absent [37]. To investigate whether the restored OCRL is physiologically functional, we measured intracellular PI(4,5)P_2_ levels as a functional indicator of OCRL activity.

Immunofluorescence imaging revealed colocalization of PI(4,5)P_2_ with the lysosomal marker LAMP1 in the Lowe patient iHTM cell line (OCRL p.D451N) (mean = 37.78%), which was significantly elevated relative to iHTM controls (mean = 18.91%) (Figure 5G). In ABE‐mediated mutation‐corrected clones (Lowe + ABE1, ABE2, and ABE3), this abnormal colocalization was robustly rescued (mean = 16.35%, 15.15%, and 11.17%, respectively). Quantification of the LAMP1‐PI(4,5)P_2_ overlap confirmed that all mutation‐corrected lines exhibited a highly significant decrease in lysosomal phosphoinositide accumulation, bringing the levels comparable to control values (Figure 5H). The results were further confirmed by functional assessment of OCRL activity in iHTM, as measured by phosphate concentration in each reaction. The Lowe patient cell (OCRL p.D451N) exhibited a profound impairment in 5‐phosphatase activity (13.8 µM). Remarkably, genetic correction via base editing successfully restored OCRL catalytic function. All three corrected clones (Lowe + ABE1, ABE2, and ABE3) demonstrated a statistically significant increase in released phosphate (26.0, 28.5, and 29.2 µM, respectively), successfully recovering baseline enzymatic activity to levels comparable with both the iHTM (30.5 µM) and pHTM wild‐type controls (30.8 µM). In addition, we investigated the cytoskeletal organization and primary cilia length in the patient cell line, iHTM control, and mutation‐corrected cell lines. Cytoskeletal organization was assessed by immunostaining for α‐actinin and F‐actin. Quantitative analysis showed that α‐actinin puncta density was markedly elevated in unedited Lowe iHTM cells compared with control iHTM cells (27.15 vs. 4.16 puncta per 100 µm^2^). Correction of the OCRL p.D451N variant substantially reduced α‐actinin puncta density to 5.91, 6.07, and 5.98 puncta per 100 µm^2^ in the ABE1, ABE2, and ABE3 groups, respectively, approaching the control level (Figure 5J). Similarly, F‐actin fiber density was strongly reduced in unedited Lowe iHTM cells compared with control cells (0.97 vs. 2.87 fibers per 100 µm^2^), whereas ABE correction increased F‐actin fiber density to 2.63, 2.55, and 2.67 fibers per 100 µm^2^ in the ABE1, ABE2, and ABE3 groups, respectively (Figure 5K). Together, these data demonstrate that correction of the OCRL p.D451N variant largely restores α‐actinin and F‐actin organization toward the control phenotype. Having established that adenine base editing restores OCRL 5‐phosphatase activity, clears lysosomal PI(4,5)P_2_ accumulation, and normalizes the actin cytoskeleton, we next investigated whether this functional rescue translates to the correction of downstream cellular phenotypes, specifically primary ciliogenesis. We performed immunofluorescence staining using ARL13B (green) and γ‐tubulin (red) to visualize the ciliary axoneme and basal bodies, respectively (Figure 5L). Consistent with the disruption of ciliogenesis typically induced by OCRL deficiency, the uncorrected Lowe patient cells exhibited a statistically significant decrease in cilium length compared to the iHTM control cells (4.01 µm vs 4.65 µm). Remarkably, genetic correction via base editing fully rescued this defect. Quantitative analysis of the violin plots confirmed that all three independent corrected clones (Lowe + ABE1, ABE2, and ABE3) demonstrated a robust and highly significant increase in cilium length relative to the uncorrected mutant line (5.04, 4.90, and 4.96 µm, respectively), restoring cilium length to levels comparable to or slightly greater than those observed in control iHTM cells (Figure 5M). The results demonstrate efficient correction of the OCRL p.D451N mutation via adenine base editing, which restores OCRL protein expression, enzymatic activity, and primary cilia length. This genetic correction improved cytoskeletal organization in patient‐derived iHTM cells, indicating that CRISPR‐mediated base editing represents a potential therapeutic strategy for patient‐specific mutations in Lowe syndrome.

### 2.5 Transcriptomic Pathway Analysis Reveals ECM‐Adhesion‐Cytoskeletal Dysregulation in Lowe iHTM Cells and Its Modulation by Genetic Correction

**FIGURE 6:**
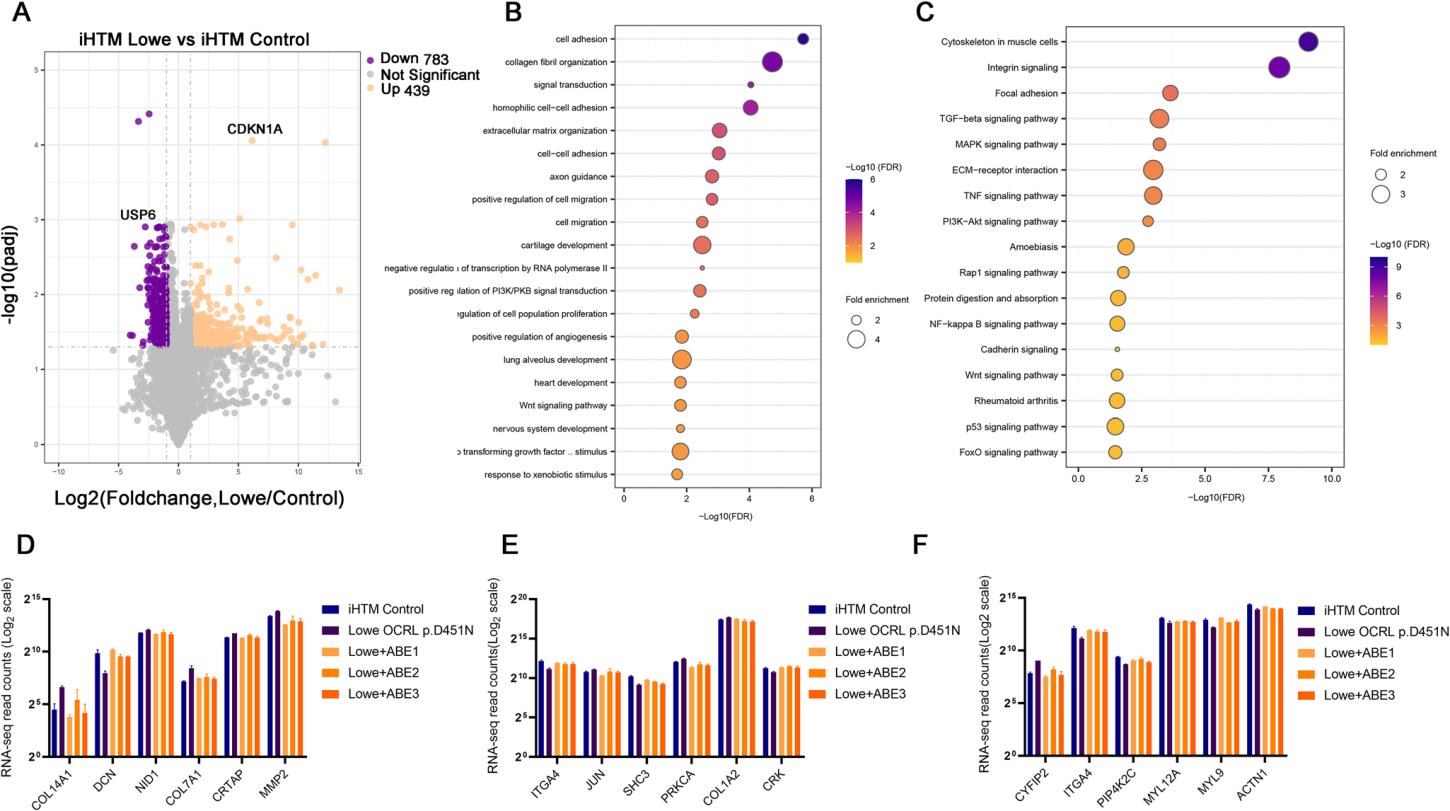
Functional enrichment analysis of bulk RNA sequencing. Volcano plot analysis revealed distinct transcriptional differences between Lowe syndrome iHTM samples (n = 8) and iHTM Control samples (n = 2) (adjusted P ≤ 0.05, |log_2_ fold change| ≥ 1). (B) The significantly enriched GO terms between the Lowe vs control comparison. (C) Dot plots show enriched KEGG pathways for Lowe syndrome iHTM and iHTM control. Dot size represents the fold enrichment of genes contributing to each pathway, and color indicates the adjusted *p*‐value. (D‐F) Differentially expressed markers in terms related to the ECM‐adhesion‐cytoskeletal network in iHTM Controls, Lowe iHTM cells, and colonies after CRISPR‐mediated mutation correction (n = 6).

Severe glaucoma with progressive irreversible blindness is observed in about half of Lowe syndrome patients [3, 38, 39]. Having firmly established the core cytoskeletal and ciliary phenotypes of our OCRL‐mutant lines and demonstrated their susceptibility to therapeutic rescue, we next sought to utilize unbiased RNA‐sequencing as a global exploratory approach to explore the potential downstream transcriptional landscape driving Lowe syndrome‐associated glaucoma pathology. We performed RNA sequencing on iHTM cells harboring Lowe syndrome‐causing mutations and iHTM Controls, followed by analyzing transcriptional differences between those groups. The principal component analysis (PCA) showed control iHTM samples generally occupied a distinct region of the PCA space compared with the Lowe syndrome‐derived iHTM samples, while the different Lowe syndrome cell lines showed some degree of heterogeneity (Figure S6A). We conducted differential expression analysis to identify distinct transcriptional changes associated with Lowe syndrome. Comparison between Lowe syndrome samples and controls revealed 439 upregulated and 783 downregulated differentially expressed genes, reflecting widespread transcriptional alterations associated with *OCRL* deficiency. (Figure 6A). Gene Ontology (GO) functional enrichment analysis of the differentially expressed genes (DEGs) between Lowe mutant and control cells revealed differences in biological processes associated with collagen fibril organization, extracellular matrix organization, and cell adhesion (Figure 6B). KEGG pathway analysis further confirmed that Lowe syndrome iHTM cells exhibit significant enrichment of pathways related to extracellular matrix‐receptor interaction, integrin signaling, focal adhesion, and multiple intracellular signaling pathways, including PI3K‐Akt, a pathway known to be regulated by *OCRL*, as well as TGF‐β signaling, when compared with healthy control cells (Figure 6C). Together, these findings indicate broad dysregulation of ECM‐, adhesion‐, and cytoskeleton‐associated transcriptional programs in OCRL‐deficient iHTM cells. To investigate whether CRISPR‐mediated mutation correction restores expression profiles in Lowe syndrome iHTMs, we next examined transcriptomic changes following genetic correction of the OCRL mutation. PCA of the RNA‐seq profiles showed that the uncorrected Lowe syndrome samples carrying the *OCRL* mutations clustered separately from the control samples, whereas the ABE‐corrected samples shifted toward the control transcriptomic space (Figure S6B). GO enrichment analysis of differentially expressed genes between uncorrected and rescued Lowe iHTM cells again highlighted biological processes associated with cell adhesion, extracellular matrix organization, cell–cell adhesion, regulation of actin cytoskeleton organization, cell migration, small GTPase signaling, and TGF‐β receptor signaling (Figure S6C). KEGG analysis similarly identified prominent enrichment of pathways including Rap1, MAPK, PI3K‐Akt, focal adhesion, integrin signaling, TGF‐β, Wnt, and regulation of the actin cytoskeleton (Figure S6D). Notably, many of these pathways overlapped with those altered between Lowe syndrome and control iHTM cells, suggesting that genetic correction preferentially affects disease‐associated ECM‐adhesion‐cytoskeletal signaling networks. These transcriptomic findings are consistent with the cytoskeletal phenotypes observed by immunofluorescence, including altered α‐actinin puncta and F‐actin fiber organization in Lowe iHTM cells and their improvement following OCRL correction. Based on the PCA, GO, and KEGG analyses, we next examined representative genes associated with extracellular matrix organization, focal adhesion/integrin signaling, and actin cytoskeletal regulation. Several disease‐associated genes showed expression changes in Lowe OCRL p.D451N iHTM cells that were shifted toward control levels following ABE‐mediated correction. Within the ECM‐related group, DCN was reduced in Lowe cells and increased toward control levels in the ABE‐corrected clones, whereas *COL14A1*, *NID1*, *COL7A1*, *CRTAP*, and *MMP2* showed Lowe‐associated increases that were partially reversed after correction (Figure 6D). Similarly, genes involved in adhesion and signaling, including *ITGA4*, *JUN*, *SHC3*, *PRKCA*, *COL1A2*, and *CRK*, showed directional changes toward the control expression pattern following ABE correction (Figure 6E). Genes associated with actin cytoskeletal regulation, including *CYFIP2*, *ITGA4*, *PIP4K2C*, *MYL12A*, *MYL9*, and *ACTN1*, also exhibited partial normalization toward control levels in the corrected cells (Figure 6F).

Collectively, the data support disruption of an interconnected ECM‐cell adhesion‐actin cytoskeleton axis as a prominent molecular feature of OCRL‐deficient iHTM cells and identify this network as responsive to genetic correction.

## 3 Discussion

To develop an appropriate model to study glaucoma in Lowe syndrome, we generated iPSC‐derived HTM‐like cells (iHTM) and proved that it is a reliable model to study Lowe syndrome. In addition, we explored a general therapeutic strategy for Lowe syndrome by evaluating three AAV vectors (AAV9‐OCRL, AAV2.3m‐OCRL, and AAV2‐OCRL) for DNA augmentation therapy. We found that AAV9‐OCRL and AAV2‐OCRL outperformed AAV2.3m‐OCRL in restoring OCRL expression and cellular phenotypes in Lowe syndrome models. This likely stems from differences in cellular tropism and receptor‐binding dynamics. Wild‐type AAV2 and AAV9 possess broad, robust tropism profiles driven by the widespread expression of their primary attachment receptors; AAV2 binds to cell‐surface heparan sulfate proteoglycans (HSPG) [40], whereas AAV9 utilizes ubiquitous α‐galactose residues. The high efficiency of these parental serotypes suggests that their native glycan receptors are highly accessible in our specific model system. Conversely, AAV2.3m is a modified capsid variant harboring triple tyrosine mutation (Y444+500+730F) designed to alter intracellular processing and narrow vector tropism toward specific target cell types like mesenchymal populations [41]. These surface modifications can induce minor topological shifts that impair general co‐receptor engagement; AAV2.3m exhibits considerably restricted transduction efficiency in cell populations where its specialized tropism is not explicitly favored. In parallel, we developed CRISPR‐based patient‐specific gene therapy for a Lowe patient, which restored OCRL protein expression and enzyme activity and rescued the cellular phenotypes. Transcriptomic analysis of iHTM models revealed dysregulation of extracellular matrix pathways, alterations in cell adhesion and signaling‐related pathways, providing insight into potential mechanisms underlying glaucoma in Lowe syndrome patients. The enriched pathways in our RNA‐seq dataset perfectly match the known pathophysiology of OCRL [33].

iHTM cells serve as a disease‐relevant human model for Lowe syndrome, enabling direct investigation of *OCRL*‐associated phenotypes. Unlike animal models of Lowe syndrome that do not replicate ocular phenotypes [4, 6, 42] or non‐ocular cell systems, iHTM cells provide a physiologically relevant platform to study possible glaucoma‐related mechanisms in a patient‐specific genetic context. Unlike dual‐knockout mouse models (Ocrl^−/−^, Inpp5b^−/−^), which result in embryonic lethality and require humanized INPP5B rescue transgenes to survive, our human‐derived cell models carrying patient‐specific nonsense mutations successfully recapitulate key cellular hallmarks of the disease under native expression conditions. This highlights the severe biological limitations of forced double‐knockout rodent lines and reinforces the physiological relevance of utilizing human genetic models to study Lowe syndrome‐associated glaucoma.

In addition, iHTM models enabled systematic comparison of therapeutic strategies, including AAV‐mediated *OCRL* DNA augmentation. Notably, direct evaluation of multiple AAV vectors identified AAV9‐OCRL and AAV2‐OCRL as more effective than AAV2.3m‐OCRL in restoring OCRL expression and cellular phenotypes in the iHTM model, highlighting their potential for Lowe syndrome treatment. In addition, the iHTM models harboring OCRL p.R541X, p.R822X, and p.R844X represent nonsense mutations. These models are suitable for evaluating nonsense readthrough therapies. As proof of concept, we treated them with the established readthrough agent PTC124 (Ataluren; ApexBio, Cat. #A8553) [43, 44] at concentrations ranging from 3 to 20 µM. However, no significant restoration of OCRL protein expression was detected compared with DMSO‐treated controls (data not shown). Nonetheless, from a translational standpoint, pharmacological approaches to premature stop codons in OCRL are highly attractive from a translational standpoint due to their established safety windows and straightforward oral administration. By reading through premature termination codons during translation, these agents can partially recover full‐length functional OCRL without altering the patient's genome.

Nascent genetic strategies present powerful, permanent alternatives that warrant consideration. For instance, adenine base editors represent a highly viable strategy for permanently correcting the recurrent A.T to G.C transitions that underlie many nonsense mutations in Lowe syndrome patients, achieving precise repair without the genomic instability linked to double‐stranded breaks. For mutations where base editing is structurally unfeasible, DNA augmentation via viral delivery of a wild‐type OCRL transgene offers a mutation‐agnostic alternative. While gene supplementation circumvents the need for patient‐specific guides, its application in Lowe syndrome remains complicated by the strict spatial and temporal regulation required for endogenous OCRL expression, potential toxicity from overexpressing the phosphatase, and the packaging constraints of standard viral vectors. Ultimately, balancing the ready availability of translational small molecules against the definitive corrective capacity of base editing or gene addition will shape the future landscape of Lowe syndrome therapeutics.

Despite their therapeutic promise, both AAV‐mediated *OCRL* gene augmentation and CRISPR‐based mutation correction have limitations. Lowe syndrome is a multiorgan disorder. Although DNA augmentation offers a mutation‐agnostic therapeutic strategy, the tissue tropism of a single AAV vector may not adequately target all affected organs, thereby limiting therapeutic efficacy in vivo. In contrast, CRISPR‐mediated correction enables precise repair of pathogenic mutations at the endogenous locus but currently faces substantial translational challenges, including delivery efficiency. In the context of this study, both strategies were evaluated in vitro using iHTM models, and their relative advantages and limitations underscore the need for careful optimization and complementary approaches when considering future therapeutic development for Lowe syndrome.

## 4 Materials and Methods

### 4.1 Plasmid Construction

SpG‐ABE8e, SpRY‐ABE8e, BE4max, and Prime Editor (PE) Plasmids Were Obtained From Addgene (#185911 and #185671, # 112093, # 174820, respectively)

### 4.2 Cell Culture and DNA Transfection

#### 4.2.1 Primary Fibroblasts

Lowe patient fibroblasts were cultured in DMEM/F‐12 (11‐330‐057; Gibco) supplemented with 10% FBS (F7524; Sigma‐Aldrich), 1% fibroblast growth supplement (Catalog # 2352, ScienCell), 1 mM GlutaMAX (Life Technologies), 100 U/mL penicillin, and 100 ug/mL streptomycin, and were incubated at 37°C with 5% CO2. Genomic DNA was extracted using One Step Mouse Genotyping Kit PD101 (Vazyme) according to the manufacturer's instructions. Sequences of primers used to confirm the *OCRL* mutation in the Lowe patient are listed in Table S1. Sanger sequencing results were analyzed by EditR [45].

#### 4.2.2 Primary Human Trabecular Meshwork Cells

Primary human trabecular meshwork cells were maintained in α‐MEM (Gibco; 12‐571‐063) supplemented with 10% heat‐inactivated fetal bovine serum (Sigma‐Aldrich; F7524) and 0.2% Primocin (InvivoGen; ant‐pm‐05) and cultured at 37°C in a humidified atmosphere with 5% CO_2_.

### 4.3 iPSC

A healthy iPSC control was dissociated into single cells using Accutase (Catalog #07920, Stemcell Technologies). 2 × 10^5^ cells were electroporated with 2.5 µg of BE4max/Spg‐ABE8e/SpRY‐ABE8e encoding plasmids and 2.5 µg of sgRNA‐encoding plasmids or 2 µg of PE encoding plasmids, 1 µg of nick RNA and 1 µg of PegRNA encoding plasmids using the Basic Nucleofector Kit (VPI‐1002; program T‐016). Cells were treated with puromycin (0.5 µg/mL) 48 h after electroporation, followed by single‐colony isolation. Control iPSCs and iPSC single colonies harboring OCRL p. R541X, OCRL p. R822X, OCRL p.R844X, OCRL p. D451N, and Lowe+ABE1/2/3 were cultured in mTeSR Plus Basal Medium (Catalog # 100–0274, Stemcell Technologies) supplemented with 1% supplement (Catalog # 100–0275, Stemcell Technologies).

### 4.4 Primary Fibroblast Reprogramming

Human conjunctival fibroblasts(P3) harboring the OCRL p. D451N mutation and a healthy control were reprogrammed under feeder‐free conditions using a non‐integrating Sendai virus system expressing the pluripotency factors OCT4, SOX2, KLF4, and c‐MYC (Catalog #A16517, Thermo Scientific). Fibroblasts were seeded into 3 wells of a 6‐well cell culture plate at a density of 1 × 10^5^ cells prior to viral exposure. On the day of transduction, cells were counted and infected with KOS, hc‐MYC, and hKLF4 Sendai vectors at MOIs of 5, 5, and 3, respectively. Following transduction, cultures were maintained in fibroblast growth medium to allow recovery and initiation of reprogramming. Seven days after transfection, cells were dissociated using Accutase (Catalog #07920, Stemcell Technologies) and transferred onto Matrigel‐coated (Catalog #08‐774‐552, Corning) 6‐well plates at densities of 2 × 10^4^ to 1 × 10^5^ cells per well. In the meanwhile, save some fibroblasts for RNA extraction to be used as a positive control in the RT‐PCR or qPCR detection of the CytoTune vector. Cultures were subsequently transitioned to mTeSR Plus (Catalog # 100–0276, Stemcell Technologies) and maintained with daily medium changes. Reprogrammed colonies with characteristic human iPSC morphology emerged over the ensuing weeks and were monitored by routine microscopic inspection. Undifferentiated colonies were identified using live‐cell staining for pluripotency‐associated surface antigens TRA‐1‐60 (Catalog# A24882, Invitrogen) and using fibroblast marker CD44 as a positive control (Catalog# A25528, Thermo Scientific). Reprogrammed colonies were manually isolated and expanded for further analysis.

### 4.5 Differentiation of iPSC Into Human Trabecular Meshwork Cells

A conditioned media approach was used to generate iHTM as reported previously [46, 47]. Briefly, the primary HTM cells were cultured in parallel with the iPSCs. The media conditioned by these primary HTM was collected daily and filtered using 0.22 µm pore filters (Corning, Catalog#431097). Subsequently, the sterile conditioned media was used to induce iPSC to differentiate, and the media was exchanged daily. iHTM were cultured using primary HTM conditioned medium up to 8 weeks. Primary HTM cells at passages ≤8 were used for experiments.

### 4.6 Immunofluorescence Staining

#### 4.6.1 iHTM and Primary HTM

Immunofluorescence staining was performed as described in our previous study [48]. Briefly, 5‐week iHTM and controls were seeded onto 4‐chamber 35 mm glass bottom (Catalog# D35C4200N, Cellvis) and grown in primary HTM‐conditioned complete media. HTM markers, α‐actinin, and F‐actin were stained simultaneously. Cells were washed three times with 1× PBS and fixed with 4% paraformaldehyde (PFA) at room temperature for 20 min, followed by three rinses with wash buffer consisting of 1× PBS containing 0.1% Triton X‐100. Samples were then incubated in a blocking solution composed of 1× PBS supplemented with 3% bovine serum albumin (BSA) and 0.1% Triton X‐100 for 45 min at room temperature. Primary antibodies were diluted in the same blocking solution and incubated with the samples overnight at 4°C, followed by three washes and incubation with the appropriate secondary antibodies for 1 h in the dark at room temperature. After secondary antibody incubation, samples were washed three additional times and counterstained with DAPI (Invitrogen) for nuclear visualization. Primary antibodies used in this study include anti‐alpha‐actinin mouse monoclonal antibody (Sigma‐Aldrich, Catalog# SAB4200813‐25UL, dilution 1:100), anti‐collagen IV antibody (Abcam, Catalog# ab6586, dilution 1:200), anti‐TIMP3 antibody (Abcam, Catalog# ab39184, dilution 1:200), anti‐myocilin antibody (Abcam, Catalog# ab318197, dilution 1:200), recombinant anti‐aquaporin 1 antibody (1:200; Abcam, ab300463), anti‐caveolin‐1 antibody (Abcam, Catalog# ab192869, dilution 1:100), anti‐VCAM1 antibody (Abcam, Catalog# ab134047, dilution 1:250), MGP Monoclonal antibody (Proteintech, Cat No. 60055‐1‐Ig), anti‐Fibronectin antibody (Abcam, Catalog# ab2413, dilution 1:200), and anti‐Laminin alpha 4/LAMA4 antibody (Abcam, Catalog# ab313467, dilution 1:200). Secondary antibodies used in this study include Goat anti‐Mouse IgM Alexa Fluor 488 (Invitrogen #A‐21042), Goat anti‐Mouse IgG (H+L) Alexa Fluor 555 (Invitrogen #A32727), Goat anti‐Rabbit IgG (H+L) Alexa Fluor 488 (Invitrogen #A11008), and Goat anti‐Rabbit IgG (H+L) Alexa Fluor 555 (Invitrogen #A21428). Samples were washed three times with PBS (5 min each) and counterstained with DAPI (Invitrogen).

### 4.7 iPSC Staining

Pluripotency of iPSC colonies was validated by immunofluorescence staining for OCT4, SSEA‐4, TRA‐1‐60, and NANOG. The staining procedure followed the same protocol used for iHTM cells, with the exception that iPSC clones were plated onto Matrigel‐coated confocal dishes and cultured overnight prior to fixation. Primary antibodies used are from Cell Signaling Technology at a 1:200 dilution, including TRA‐1‐60 mouse monoclonal antibody (#4746), NANOG rabbit polyclonal antibody (#3580), OCT4 rabbit monoclonal antibody (#2840), and SSEA‐4 mouse monoclonal antibody (#4755).

### 4.8 Live Cell Staining

Before picking iPSC colonies for expansion, the colonies were validated by live‐cell staining. Briefly, add a 1:50 volume of TRA‐1‐60 Alexa Fluor 594 (Thermo Scientific, Catalog#A24882) and CD44 Alexa Fluor 488 (Thermo Scientific, Catalog#A25528) directly to the cell culture medium of the cells to be stained. Mix by gentle swirling. Incubate for 30 min at 37°C. Remove the staining solution and gently wash the cells 2–3 times with FluoroBriteTM DMEM (Cat. No. A1896701). iPSC colonies were imaged within 30 min using a Zeiss LSM880 confocal microscope.

### 4.9 PI(4,5)P2 and LAMP1 Staining

PI(4,5)P_2_ and LAMP1 staining and analysis were adapted from previous studies [37, 48]. Briefly, cells were seeded onto 12 mm diameter glass coverslips and grown in complete media, followed by fixation with 4% formaldehyde, 0.01% Triton X‐100, and 0.2% glutaraldehyde at room temperature. Cells were placed on ice and chilled for 5 min after rinsing with PBS containing 50 mM NH_4_Cl. Cells were blocked and permeabilized on ice with chilled blocking buffer (5% normal goat serum, 50 mM NH_4_Cl, and 0.5% saponin (Thermo Fisher Scientific, Cat. No. 55‐825‐5100GM)) for 45 min. Cells were then stained with PIP2 antibody (PIP2 2C11) (1:400; Santa Cruz Biotechnology, Cat.No. sc‐53412) in 5% normal goat serum and 0.1% saponin for 1 h before post‐fixing cells with 2% formaldehyde for 10 min. after three washes, cells were then incubated with anti‐LAMP‐1 Antibody (H4A3) (Novus biologicals #NBP2‐25183, dilution, 1:500) overnight. Alexa 488‐conjugated goat anti‐mouse IgM antibody (1:500; Invitrogen, Cat. No. A‐21042) and goat anti‐mouse IgG1 secondary antibody were applied in the same solution for 45 min. DNA was visualized using 4,6‐diamidino‐2‐phenylindole (DAPI; Thermo Fisher Scientific) for 10 min. Images were taken under non‐saturated conditions using a confocal microscope (LSM 880 Zeiss confocal microscope) with the 63 x oil immersion objective and Z‐stacks. All images were acquired with the same settings and analyzed using ImageJ. Briefly, the level of co‐localization (PI(4,5)P_2_‐LAMP1) was calculated by acquiring confocal z‐stacks from about 25 views (each view has 3–5 cells) per experimental condition per experiment, exported in TIFF format using ZEN software. Briefly, confocal images of iHTM Control, Lowe (OCRL p.D451N), and Lowe+ABE1/2/3 were acquired at the same laser power and photomultiplier gain. Images were then processed using ImageJ software. TIFF images were split into single channels from each image and were converted into 8‐bit greyscale images before being thresholded to subtract background. The ImageJ ‘Analyze Particles’ plugin was then used to identify and count the total number of structures (with an area above 0.03 µm^2^) in the LAMP1 channel. The structures in the PI(4,5)P_2_ channel were used to build a mask that was then used in the “Image Calculator” to count PI(4,5)P_2_ ‐LAMP1 positive structures, which was then divided by the total number of LAMP1‐positive structures.

### 4.10 Quantification of F‐Actin

F‐actin fibers were quantified from phalloidin‐stained fluorescence images using the Ridge Detection plugin in Fiji/ImageJ, as previously described [49, 50]. Cell boundaries were drawn manually using the freehand tool, and cell area was measured using the ROI Manager tool. Fibers were detected using the following Ridge Detection parameters: line width, 20.0; high contrast, 230; low contrast, 100; sigma, 6.57; lower threshold, 0.00; and upper threshold, 0.34. The same acquisition and analysis parameters were applied to all experimental groups. F‐actin fiber density was quantified as the total number of detected fibers normalized to cell area and expressed as fibers per 100 µm^2^.

### 4.11 α‐Actinin Puncta Quantification

α‐Actinin puncta were quantified using the Analyze Particles function in Fiji/ImageJ. The phalloidin channel was used to manually outline individual cell boundaries, which were saved as regions of interest (ROIs) in the ROI Manager and used to measure cell area. For α‐actinin puncta detection, the α‐actinin channel was thresholded using the RenyiEntropy method. Puncta were then quantified using Analyze Particles with a minimum particle size of 0.1 µm^2^ and a circularity range of 0.2–1.0. The number of α‐actinin puncta was normalized to the corresponding cell area. α‐Actinin puncta density was expressed as the number of puncta per 100 µm^2^ of cell area.

### 4.12 Ciliogenesis Assays

Primary cilium visualization was performed according to a previously established protocol [48]. Briefly, cells cultured on 12‐mm glass coverslips were subjected to 40–48 h of serum starvation in complete medium to induce ciliogenesis. For immunostaining, cells were fixed with ice‐cold methanol at −20°C for 20 min, then blocked and permeabilized using a buffer of 1x PBS containing 3% BSA and 0.1% Triton X‐100 for 30 min at room temperature. Primary antibodies, including anti‐Arl13b (NeuroMab, #N295B/66; 1:1000) and anti‐γ‐tubulin (Abcam, ab11317; 1:500), were diluted in blocking buffer and incubated overnight at 4°C. Subsequently, cells were incubated with Alexa Fluor 488‐ and 555‐conjugated secondary antibodies (1:500; Thermo Fisher Scientific) for 1 h. Nuclei were counterstained with DAPI, and coverslips were mounted onto glass slides utilizing ProLong Gold Antifade medium. Confocal imaging was performed on a Zeiss LSM880 microscope equipped with a 63x oil immersion objective. Cilia length quantification was conducted via ImageJ, utilizing the line tool for linear cilia and the segmented line tool for curved structures.

### 4.13 Reverse Transcription PCR (RT‐PCR) and Quantitative Real‐Time PCR

Total RNA was extracted from patient cells seven days after Sendai virus transduction, P1‐P12 iPSC colonies, non‐transduced cells, Lowe (OCRL p.R541X), Lowe (OCRL p.R822X), Lowe (OCRL p.R844X) iPSC and iHTM clines or iHTM control and Lowe (OCRL p. D451N) cells using Quick‐RNA Miniprep Plus Kit (ZYMO RESEARCH, Cat. No. R1058) according to the manufacturer's instructions. cDNA was synthesized with HiScript II First Strand cDNA Synthesis Kit (Vazyme, Cat. No. R212). Primers used for RT‐PCR and qPCR are listed in Table S2.

### 4.14 DEX‐Ac Treatment

iHTM cells (iHTM controls, Lowe (OCRL p.R541X), Lowe (OCRL p.R822X), Lowe (OCRL p.R844X), OCRL p.D451N, Lowe+ABE1/2/3) were seeded at less than 10% confluence so that cell density reaches 90%‐100% confluence after 14 days of treatment with 100 nM DEX‐Ac. 2% complete medium was changed every two days.

### 4.15 Off‐Target Editing Analysis

Top 5 potential off‐target sites for each sgRNA used to generate Lowe syndrome cellular models were predicted using an online tool (http://www.rgenome.net/cas‐offinder/). Sequences of potential off‐target sites and primers used to amplify target sequences are listed in Table S3. Genomic DNA was extracted from iHTM Control and Lowe (OCRL p.R541X, OCRL p.R822X, OCRL p.R844X) for off‐target editing analysis. The PCR products were subjected to Sanger sequencing and then analyzed by EditR.

### 4.16 Western Blotting

Cells were collected and homogenized in RIPA Lysis Buffer (Millipore Sigma, Cat. No. 20–188) for western blotting. The protein concentrations were measured using the PIERCE BCA protein assay kit (Thermo Scientific, Cat. No. 23227). A total of 20 µg of each cell line was loaded. Anti‐OCRL antibody (1:250; Sigma‐Aldrich HPA012495‐100UL), myocilin Antibody (F‐12) (1:800; Santa Cruz Biotechnology, Cat. No. sc‐137233) and anti‐α‐tubulin polyclonal antibody (1:5000; Proteintech, Cat. No. 11224‐1‐AP) were used as primary and internal controls, respectively. Signals were acquired by direct measurement of chemiluminescence using a digital camera (Amersham Imager 600), and quantification was done using ImageJ software from at least three independent experiments [48].

### 4.17 AAV‐OCRL Transduction

AAV9‐OCRL, AAV2.3m‐OCRL, and AAV2‐OCRL were purchased from AAVnerGene. Five‐week‐old iHTM cells harboring the OCRL p.D451N mutation were seeded into 6‐well plates to reach approximately 70% confluence the following day. Cells were transduced with AAV9‐OCRL, AAV2.3m‐OCRL, or AAV2‐OCRL at a multiplicity of infection (MOI) of 1 × 10^5^. Six days after transduction, cells were either harvested for protein extraction or reseeded onto confocal dishes for phenotypic analysis.

### 4.18 OCRL Phosphatase Activity Assay

OCRL phosphatase activity was quantified using a malachite green phosphate assay kit (Sigma‐Aldrich, Catalog# MAK307‐1KT) to measure inorganic phosphate (Pi) release. Briefly, six‐week‐old iHTM cells and primary HTM were prepared in phosphate‐free assay buffer (50 mM Tris‐HCl, pH 7.5, 2 mM MgCl_2_, 0.1% Triton X‐100 in ultra‐pure water). Reactions were performed in 96‐well plates with 10 µg cell lysate (60 µL) and 20 µL PI(4,5)P2 diC8 substrate (Echelon Biosciences, Catalog# P‐4508) in a total volume of 80 µL and incubated at 37°C for 30 min. Reactions were terminated by adding 20 µL of Malachite Green reagent, followed by 25 min incubation at room temperature, protected from light. Absorbance was measured at 620 nm, and Pi release was calculated using a standard curve (0–60 µM). OCRL activity was presented using free phosphate concentration in the reactions. All reactions were performed in technical triplicates.

### 4.19 Bulk RNA Sequencing Analysis

Six weeks after differentiation, total RNA was isolated from two iHTM controls, eight Lowe syndrome iHTM cells, and six mutation‐corrected colonies using TRIzol reagent following the manufacturer's protocol. Bulk RNA sequencing was performed by Novogene (California, USA). Raw sequencing reads were quality‐filtered using SOAP to obtain clean reads, which were subsequently aligned to the reference transcriptome with HISAT2. Clean reads were also mapped to the reference gene set using Bowtie2, and transcript abundance was quantified with RSEM, expressed as fragments per kilobase of exon per million mapped reads (FPKM). Statistical differences in genes were analyzed using the R stat (v.4.1.2) package using Student's *t*‐test (two‐sided) and followed by the Benjamini‐Hochberg (BH) multiple comparisons test (FDR). Genes with FDR‐corrected *P* < 0.05 and FC >2 or <0.5 were considered differentially expressed genes (DEGs) and used for subsequent analysis. Functional enrichment analyses, including Gene Ontology (GO) and Kyoto Encyclopedia of Genes and Genomes (KEGG) pathway analyses, were conducted based on the identified DEGs using the Database for Annotation, Visualization and Integrated Discovery (https://davidbioinformatics.nih.gov) with Fisher's exact test followed by the BH multiple comparison test as FDR‐corrected P < 0.05, as mentioned in the previous study [51].

### 4.20 Principal Component Analysis (PCA)

RNA‐seq count data were log2‐transformed as log2(count + 1) prior to principal component analysis. PCA was performed using SIMCA 18.2 (Sartorius Stedim Data Analytics AB, Umeå, Sweden), with samples defined as observations and genes as X variables. PCA‐X models were generated using unit‐variance (UV) scaling, and score plots were used to visualize similarities and differences in global transcriptional profiles among samples.

### 4.21 Statistical Analysis

All data are expressed as mean ± SEM and represent at least three individual determinations in all experiments. The data were analyzed using one‐way ANOVA followed by Dunnett's multiple‐comparisons test or the Student's *t*‐test using GraphPad Prism software 8. The probability of p < 0.05 was considered statistically significant (n.s., not significant, ^*^ *p* < 0.05, ^**^ *p* < 0.01, ^***^ *p* < 0.001, ^****^ *p* < 0.0001).

## Author Contributions

S.C. and Z.L. performed the experiments and analyzed the data. T.J.K. contributed to iHTM differentiation. W.W. contributed to RNA‐seq data analysis. Q.W. contributed AAV‐OCRL viruses; H.M.L., F.Z., G.W., F.H., J.Z. and Y.W. contributed reagents/materials/analysis tools. S.C. wrote the paper. Y.S. supervised the whole project. All authors have read and approved the manuscript.

## Conflicts of Interest

The authors declare no conflicts of interest.

## Supporting information




**Supporting File 1**: advs77704‐sup‐0001‐SuppMat.pdf.

**Supporting File 2**: advs77704‐sup‐0002‐SuppMat.xlsx.

## Data Availability

Data supporting the findings of this study are available in the Article and Supporting Information. RNA‐seq data have been deposited in the National Center for Biotechnology Information (NCBI) Sequence Read Archive (SRA) database with BioProject accession code PRJNA1422933.

## References

[1] A. Bökenkamp and M. Ludwig , “The Oculocerebrorenal Syndrome of Lowe: An Update,” Pediatric Nephrology 31, no. 12 (2016): 2201–2212, 10.1007/s00467-016-3343-3. 27011217 PMC5118406

[2] M. Loi and L. syndrome , “Lowe Syndrome,” Orphanet Journal of Rare Diseases 1, no. 1, (2006): 16, 10.1186/1750-1172-1-16. 16722554 PMC1526415

[3] X. Ma , K. Ning , and S. Jabbehdari , “Oculocerebrorenal Syndrome of Lowe: Survey of Ophthalmic Presentations and Management,” European Journal of Ophthalmology 30, no. 5 (2020): 966–973, 10.1177/1120672120920544. 32340490 PMC8177091

[4] S. P. Bothwell , E. Chan , I. M. Bernardini , Y.‐M. Kuo , W. A. Gahl , and R. L. Nussbaum , “Mouse Model for Lowe Syndrome/Dent Disease 2 Renal Tubulopathy,” Journal of the American Society of Nephrology 22, no. 3 (2011): 443–448, 10.1681/ASN.2010050565. 21183592 PMC3060438

[5] B. E. Kadhi , et al., “The Inositol 5‐phosphatase dOCRL Controls PI(4,5)P2 Homeostasis and Is Necessary for Cytokinesis,” Current Biology 21, no. 12 (2011): 1074–1079. 21658948 10.1016/j.cub.2011.05.030

[6] I. B.‐R. Ramirez , G. Pietka , and D. R. Jones , “Impaired Neural Development in a Zebrafish Model for Lowe Syndrome,” Human Molecular Genetics 21, no. 8 (2012): 1744–1759, 10.1093/hmg/ddr608. 22210625 PMC3313792

[7] B. G. Coon , V. Hernandez , and K. Madhivanan , “The Lowe Syndrome Protein OCRL1 Is Involved in Primary Cilia Assembly,” Human Molecular Genetics 21, no. 8 (2012): 1835–1847, 10.1093/hmg/ddr615. 22228094

[8] P. Zhu , “Optogenetic Dissection of Neuronal Circuits in Zebrafish Using Viral Gene Transfer and the Tet System,” Frontiers in Neural Circuits 3 (2009): 21, 10.3389/neuro.04.021.2009. 20126518 PMC2805431

[9] W.‐C. Hsieh , S. Ramadesikan , D. Fekete , and R. C. Aguilar , “Kidney‐differentiated Cells Derived From Lowe Syndrome Patient's iPSCs Show Ciliogenesis Defects and Six2 Retention at the Golgi Complex,” PLoS ONE 13, no. 2 (2018): 0192635, 10.1371/journal.pone.0192635. PMC5812626 29444177

[10] B. M. Akhtar , P. Bhatia , and S. Acharya , “A human Stem Cell Resource to Decipher the Biochemical and Cellular Basis of Neurodevelopmental Defects in Lowe syndrome,” Biology Open 11, no. 1 (2022): bio059066, 10.1242/bio.059066. 35023542 PMC8822356

[11] M. Berquez , J. R. Gadsby , and B. P. Festa , “The phosphoinositide 3‐kinase inhibitor alpelisib restores actin organization and improves proximal tubule dysfunction in vitro and in a mouse model of Lowe syndrome and Dent disease,” Kidney International 98, no. 4 (2020): 883–896, 10.1016/j.kint.2020.05.040. 32919786 PMC7550850

[12] K. Madhivanan , S. Ramadesikan , and W.‐C. Hsieh , “Lowe syndrome Patient Cells Display mTOR‐ and RhoGTPase‐dependent Phenotypes Alleviated by Rapamycin and Statins,” Human Molecular Genetics 29, no. 10 (2020): 1700–1715, 10.1093/hmg/ddaa086. 32391547 PMC7322567

[13] V. Miraldi Utz , R. G. Coussa , F. Antaki , and E. I. Traboulsi , “Gene Therapy for RPE65‐related Retinal Disease,” Ophthalmic Genetics 39, no. 6 (2018): 671–677, 10.1080/13816810.2018.1533027. 30335549

[14] J. R. Mendell , F. Muntoni , and C. M. McDonald , “AAV Gene Therapy for Duchenne Muscular Dystrophy: The EMBARK Phase 3 Randomized Trial,” Nature Medicine 31, no. 1 (2025): 332–341, 10.1038/s41591-024-03304-z. PMC11750718 39385046

[15] J. Lv , H. Wang , and X. Cheng , “AAV1‐hOTOF Gene Therapy for Autosomal Recessive Deafness 9: A Single‐arm Trial,” The Lancet 403, no. 10441 (2024): 2317–2325, 10.1016/S0140-6736(23)02874-X. 38280389

[16] M. F. Richter , K. T. Zhao , and E. Eton , “Phage‐assisted Evolution of an Adenine Base Editor With Improved Cas Domain Compatibility and Activity,” Nature Biotechnology 38, no. 7 (2020): 883–891, 10.1038/s41587-020-0453-z. PMC7357821 32433547

[17] A. Lapinaite , G. J. Knott , and C. M. Palumbo , “DNA Capture by a CRISPR‐Cas9–Guided Adenine Base Editor,” Science 369, no. 6503 (2020): 566–571, 10.1126/science.abb1390. 32732424 PMC8598131

[18] O. O. Abudayyeh , J. S. Gootenberg , and B. Franklin , “A Cytosine Deaminase for Programmable Single‐base RNA Editing,” Science 365, no. 6451 (2019): 382–386, 10.1126/science.aax7063. 31296651 PMC6956565

[19] A. V. Anzalone , P. B. Randolph , and J. R. Davis , “Search‐and‐replace Genome Editing Without Double‐strand Breaks or Donor DNA,” Nature 576, no. 7785 (2019): 149–157, 10.1038/s41586-019-1711-4. 31634902 PMC6907074

[20] N. M. Gaudelli , A. C. Komor , and H. A. Rees , “Programmable Base Editing of A•T to G•C in Genomic DNA Without DNA Cleavage,” Nature 551, no. 7681 (2017): 464–471, 10.1038/nature24644. 29160308 PMC5726555

[21] K. Musunuru , S. A. Grandinette , and X. Wang , “Patient‐Specific in Vivo Gene Editing to Treat a Rare Genetic Disease,” New England Journal of Medicine 392, no. 22 (2025): 2235–2243, 10.1056/NEJMoa2504747. 40373211 PMC12713542

[22] J. Kim , C. Hu , and C. Moufawad El Achkar , “Patient‐Customized Oligonucleotide Therapy for a Rare Genetic Disease,” New England Journal of Medicine 381, no. 17 (2019): 1644–1652, 10.1056/NEJMoa1813279. 31597037 PMC6961983

[23] J. Buffault , A. Labbé , P. Hamard , F. Brignole‐Baudouin , and C. Baudouin , “The Trabecular Meshwork: Structure, Function and Clinical Implications. A Review of the Literature,” Journal Français d'Ophtalmologie 43, no. 7 (2020): e217–e230, 10.1016/j.jfo.2020.05.002. 32561029

[24] B. M. Braunger , R. Fuchshofer , and E. R. Tamm , “The Aqueous Humor Outflow Pathways in Glaucoma: A Unifying Concept of Disease Mechanisms and Causative Treatment,” European Journal of Pharmaceutics and Biopharmaceutics 95, (2015): 173–181, 10.1016/j.ejpb.2015.04.029. 25957840

[25] L. Zhang , S. Wang , R. Mao , et al., “Genotype‐Phenotype Correlation Reanalysis in 83 Chinese Cases With OCRL Mutations,” Genetics Research (Camb) 2022, (2022): 1473260. 10.1155/2022/1473260 PMC9325342 35919034

[26] W. Röschinger , A. C. Muntau , G. Rudolph , A. A. Roscher , and S. Kammerer , “Carrier Assessment in Families With Lowe Oculocerebrorenal Syndrome: Novel Mutations in the OCRL1 Gene and Correlation of Direct DNA Diagnosis With Ocular Examination,” Molecular Genetics and Metabolism 69, no. 3 (2000): 213–222, 10.1006/mgme.1999.2955. 10767176

[27] H. Hichri , J. Rendu , and N. Monnier , “From Lowe Syndrome to Dent Disease: Correlations Between Mutations of the OCRL1 Gene and Clinical and Biochemical Phenotypes,” Human Mutation 32, no. 4 (2011): 379–388, 10.1002/humu.21391. 21031565

[28] W. Zhu , C. R. Godwin , L. Cheng , T. E. Scheetz , and M. H. Kuehn , “Transplantation of iPSC‐TM Stimulates Division of Trabecular Meshwork Cells in human Eyes,” Scientific Reports 10, no. 1 (2020): 2905, 10.1038/s41598-020-59941-0. 32076077 PMC7031365

[29] J. R. Polansky , D. J. Fauss , and C. C. Zimmerman , “Regulation of TIGR/MYOC Gene Expression in human Trabecular Meshwork Cells,” Eye 14, no. 3 (2000): 503–514, 10.1038/eye.2000.137. 11026980

[30] F. Langer , M. Binter , and X. Hu , “In Vitro Comparison of human and Murine Trabecular Meshwork Cells: Implications for Glaucoma Research,” Scientific Reports 14, no. 1 (2024): 22002, 10.1038/s41598-024-73057-9. 39313534 PMC11420201

[31] A. de Sa , G. Li , C. Byrne , et al., “The Inositol 5‐phosphatases OCRL and INPP5B: Cellular Functions and Roles in Disease,” Biochim Biophys Acta Mol Cell Biol Lipids 1870, no. 6 (2025): 159660. 40618840 10.1016/j.bbalip.2025.159660

[32] R. Montjean , R. Aoidi , and P. Desbois , “OCRL‐mutated Fibroblasts From Patients With Dent‐2 Disease Exhibit INPP5B‐independent Phenotypic Variability Relatively to Lowe syndrome Cells,” Human Molecular Genetics 24, no. 4 (2015): 994–1006, 10.1093/hmg/ddu514. 25305077

[33] Z. B. Mehta , G. Pietka , and M. Lowe , “The Cellular and Physiological Functions of the Lowe Syndrome Protein OCRL1,” Traffic 15, no. 5 (2014): 471–487, 10.1111/tra.12160. 24499450 PMC4278560

[34] N. Luo , M. D. Conwell , and X. Chen , “Primary Cilia Signaling Mediates Intraocular Pressure Sensation,” Proceedings of the National Academy of Sciences 111, no. 35 (2014): 12871–12876, 10.1073/pnas.1323292111. PMC4156748 25143588

[35] B. Wang , W. He , and P. P. Prosseda , “OCRL Regulates Lysosome Positioning and mTORC1 Activity Through SSX2IP‐mediated Microtubule Anchoring,” The EMBO Reports 22, no. 7 (2021): 52173, 10.15252/embr.202052173. PMC8406401 33987909

[36] R. T. Walton , K. A. Christie , M. N. Whittaker , and B. P. Kleinstiver , “Unconstrained Genome Targeting With near‐PAMless Engineered CRISPR‐Cas9 Variants,” Science 368, no. 6488 (2020): 290–296, 10.1126/science.aba8853. 32217751 PMC7297043

[37] M. G. De Leo , et al., “Autophagosome‐lysosome Fusion Triggers a Lysosomal Response Mediated by TLR9 and Controlled by OCRL,” Nature Cell Biology 18, no. 8 (2016): 839–850. 27398910 10.1038/ncb3386 PMC5040511

[38] S. J. Kruger , “Cataracts and Glaucoma in Patients With Oculocerebrorenal Syndrome,” Archives of Ophthalmology 121, no. 9 (2003): 1234–1237, 10.1001/archopht.121.9.1234. 12963605

[39] D. S. Walton , G. Katsavounidou , and C. U. Lowe , “Glaucoma With the Oculocerebrorenal Syndrome of Lowe,” Journal of Glaucoma 14, no. 3 (2005): 181–185, 10.1097/01.ijg.0000158850.07732.05. 15870597

[40] J. O'Donnell , K. A. Taylor , and M. S. Chapman , “Adeno‐associated virus‐2 and its primary cellular receptor—Cryo‐EM structure of a heparin complex,” Virology 385, no. 2 (2009): 434–443, 10.1016/j.virol.2008.11.037. 19144372 PMC2684845

[41] M. Li , G. R. Jayandharan , and B. Li , “High‐efficiency Transduction of Fibroblasts and Mesenchymal Stem Cells by Tyrosine‐mutant AAV2 Vectors for Their Potential Use in Cellular Therapy,” Human Gene Therapy 21, no. 11 (2010): 1527–1543, 10.1089/hum.2010.005. 20507237 PMC2987376

[42] M. Lowe , “Modelling Lowe Syndrome and Dent‐2 Disease Using Zebrafish,” Frontiers in Cell and Developmental Biology 13 (2025): 1637005, 10.3389/fcell.2025.1637005. 40778266 PMC12329224

[43] R. Perriera , E. Vitale , and I. Pibiri , “Readthrough Approach Using NV Translational Readthrough‐Inducing Drugs (TRIDs): A Study of the Possible off‐Target Effects on Natural Termination Codons (NTCs) on TP53 and Housekeeping Gene Expression,” International Journal of Molecular Sciences 24, no. 20 (2023): 15084, 10.3390/ijms242015084. 37894764 PMC10606485

[44] C. Vössing , M. Owczarek‐Lipska , K. Nagel‐Wolfrum , C. Reiff , C. Jüschke , and J. Neidhardt , “Translational Read‐Through Therapy of RPGR Nonsense Mutations,” International Journal of Molecular Sciences 21, no. 22 (2020): 8418. 33182541 10.3390/ijms21228418 PMC7697989

[45] M. G. Kluesner , D. A. Nedveck , and W. S. Lahr , “EditR: A Method to Quantify Base Editing From Sanger Sequencing,” The CRISPR Journal 1, no. 3 (2018): 239–250, 10.1089/crispr.2018.0014. 31021262 PMC6694769

[46] Q. J. Ding , W. Zhu , A. C. Cook , K. R. Anfinson , B. A. Tucker , and M. H. Kuehn , “Induction of Trabecular Meshwork Cells From Induced Pluripotent Stem Cells,” Investigative Opthalmology & Visual Science 55, no. 11 (2014): 7065–7072, 10.1167/iovs.14-14800. PMC4224582 25298418

[47] W. Zhu , A. Jain , O. W. Gramlich , B. A. Tucker , V. C. Sheffield , and M. H. Kuehn , “Restoration of Aqueous Humor Outflow Following Transplantation of iPSC‐Derived Trabecular Meshwork Cells in a Transgenic Mouse Model of Glaucoma,” Investigative Opthalmology & Visual Science 58, no. 4 (2017): 2054–2062, 10.1167/iovs.16-20672. PMC6108236 28384726

[48] S. Chen , C.‐H. Lo , and Z. Liu , “Base Editing Correction of OCRL in Lowe Syndrome: ABE‐mediated Functional Rescue in Patient‐derived Fibroblasts,” Human Molecular Genetics 33, no. 13 (2024): 1142–1151, 10.1093/hmg/ddae045. 38557732 PMC12099292

[49] R. Kumari , K. Ven , and M. Chastney , “Focal Adhesions Contain Three Specialized Actin Nanoscale Layers,” Nature Communications 15, no. 1 (2024): 2547, 10.1038/s41467-024-46868-7. PMC10957975 38514695

[50] R. Kumari , Y. Jiu , and P. J. Carman , “Tropomodulins Control the Balance Between Protrusive and Contractile Structures by Stabilizing Actin‐Tropomyosin Filaments,” Current Biology 30, no. 5 (2020): 767–778, 10.1016/j.cub.2019.12.049. 32037094 PMC7065974

[51] W. Wang , J. Wang , and K. Yao , “Metabolic Characterization of Hypertrophic Cardiomyopathy in human Heart,” Nature Cardiovascular Research 1, no. 5 (2022): 445–461, 10.1038/s44161-022-00057-1. 39195941

