# Supplementary material for: CRISPR and Gene Augmentation Rescue Trabecular Meshwork Dysfunction in iPSC Models of Lowe Syndrome
Source: Adv Sci (Weinh). 2026 Sep 9:e77704. Online ahead of print. doi: 10.1002/advs.77704 (PMC13558910; doi:10.1002/advs.77704)
Supplement: Supplementary file 1 [file ADVS-9999-e77704-s001.pdf]

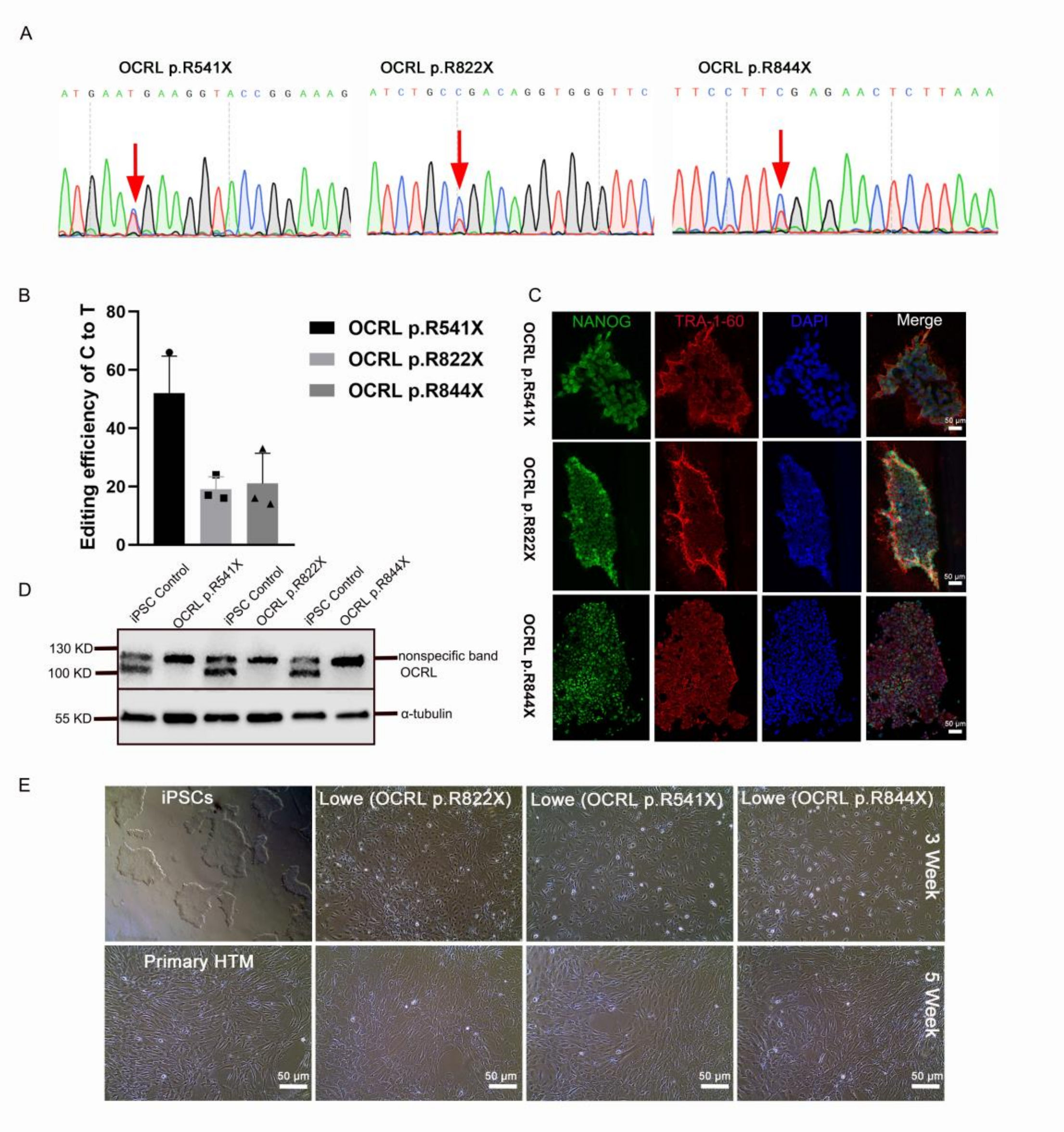

Supplementary Figure 1 **Generation of iPSC lines harboring Lowe syndrome-causing mutations in *OCRL***. (A) Representative Sanger sequencing chromatograms of bulk iPSCs demonstrating successful introduction of OCRL p.R541X, p.R822X, and p.R844X mutations by base or prime editing. Targeted nucleotides are indicated by red arrows. (B) Quantification of gene editing efficiencies at individual target sites in bulk cells. (C) Representative immunofluorescence images showing expression of pluripotency markers NANOG and TRA-1-60 in iPSCs; nuclei are counterstained with DAPI. Scale bar, 50 μm. (D) Western blot analysis showing absence of OCRL protein in iPSC lines harboring OCRL p.R541X, p.R822X, and p.R844X mutations. (E) Representative phase-contrast images showing the morphological progression of iPSCs before differentiation, primary human trabecular meshwork (HTM) cells, and iPSC lines containing distinct Lowe syndrome-causing OCRL mutations (p.R822X, p.R541X, and p.R844X). Phenotypes are tracked chronologically at 3 weeks and 5 weeks post-differentiation. Scale bar, 50 μm.

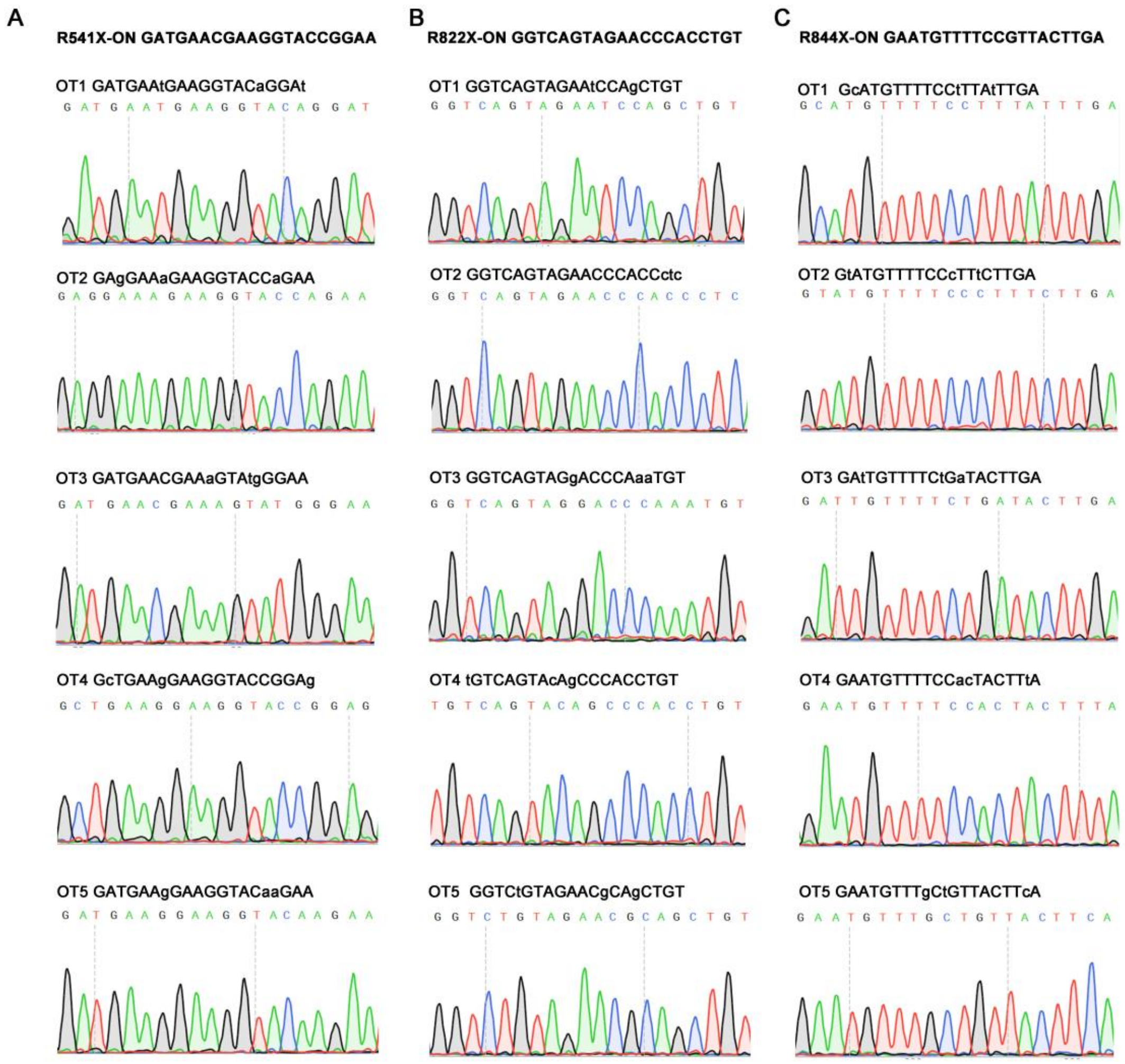

Supplementary Figure 2 **Analysis of potential off-target editing sites used for SpRY-ABE8e-mediated correction of OCRL nonsense mutations.** (A) Potential off-target (OT) sites for the sgRNAs targeting the OCRL nonsense mutations p.R541X. (B) Potential off-target (OT) sites for the sgRNAs targeting the OCRL nonsense mutations p.R822X. (C) Potential off-target (OT) sites for the sgRNAs targeting the OCRL nonsense mutations p.R844X.

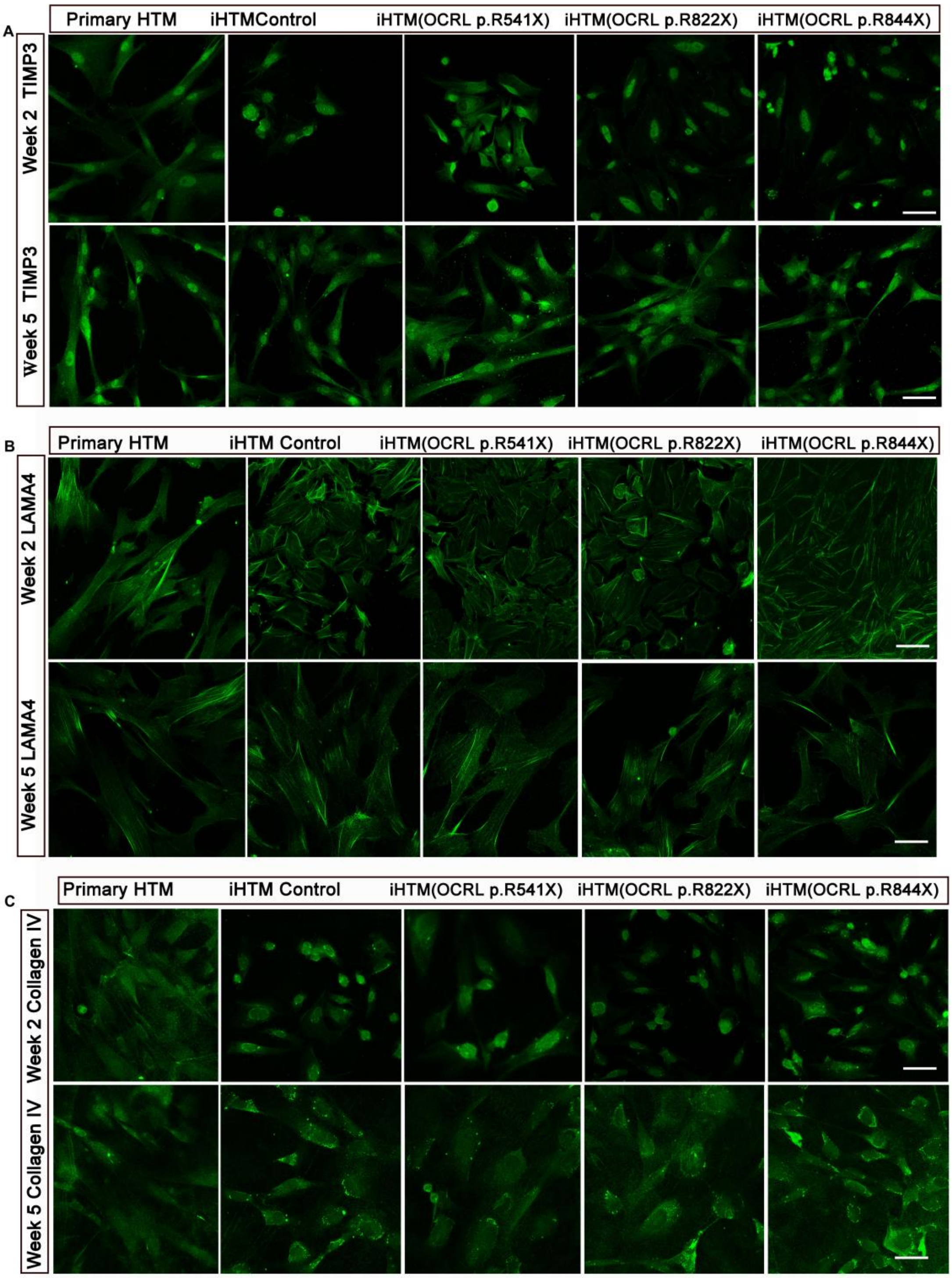

Supplementary Figure 3 **Validation of iPSC-derived trabecular meshwork cells(iHTM) with Lowe syndrome-causing mutations in *OCRL*.** (A-C) Representative immunofluorescence images showing TIMP3, Laminin, and Collagen IV staining in primary human trabecular meshwork (HTM) cells, control iPSC-derived HTM cells (iHTM Control), and iHTM cells harboring OCRL p.R541X, p.R822X, or p.R844X mutations. Cells were analyzed at week 2 (top row) and week 5 (bottom row) following differentiation. Scale bars, 50 µm.

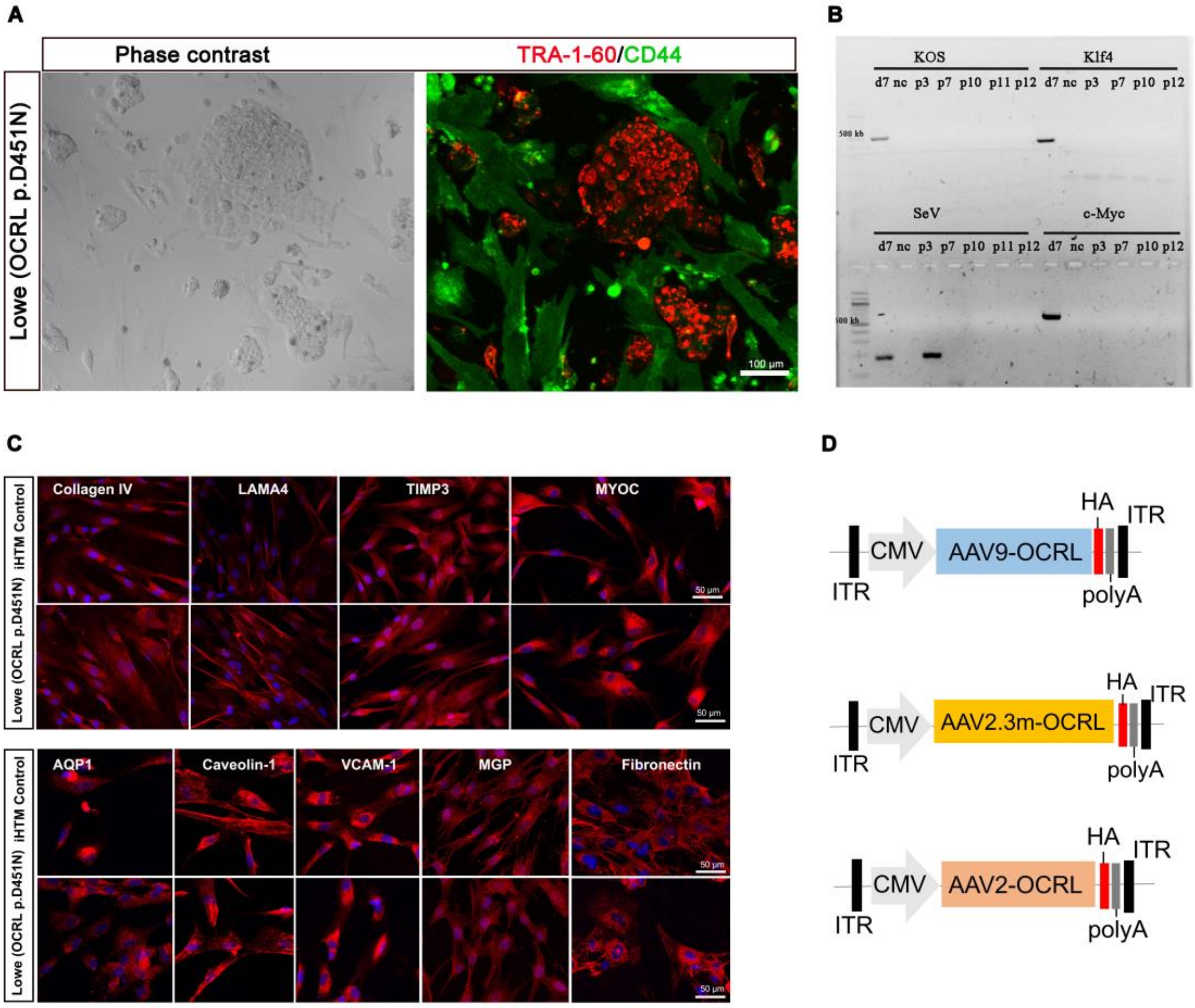

Supplementary Figure 4 **Reprogramming of Lowe Patient Fibroblasts and Differentiation into Human Trabecular Meshwork-Like Cells**. (A) Representative phase-contrast and live-cell immunofluorescence images showing Lowe (OCRL p.D451N) iPSC colonies expressing the pluripotency marker TRA-1-60 and fibroblasts expressing CD44. Scale bar, 100 µm. (B) RT-PCR analysis showing clearance of Sendai virus (SeV) reprogramming vectors (KOS, Klf4, and c-Myc) after passage 3 and complete loss of SeV signal by passage 7. d7, day 7 after transduction; P3-P12, passages 3-12 iPSCs; nc, non-transduced cells. (C) Representative immunofluorescence images showing TIMP3, Collagen IV, LAMA4, MYOC (Myocilin), AQP1 (Aquaporin-1), Caveolin-1, VCAM-1, MGP (Matrix Gla Protein), and Fibronectin staining in Control and Lowe patient iPSC-derived human trabecular meshwork (iHTM) cells. Nuclei are counterstained with DAPI. Scale bar, 50 µm. (D) Schematic diagram illustrating the AAV vectors used in this study.

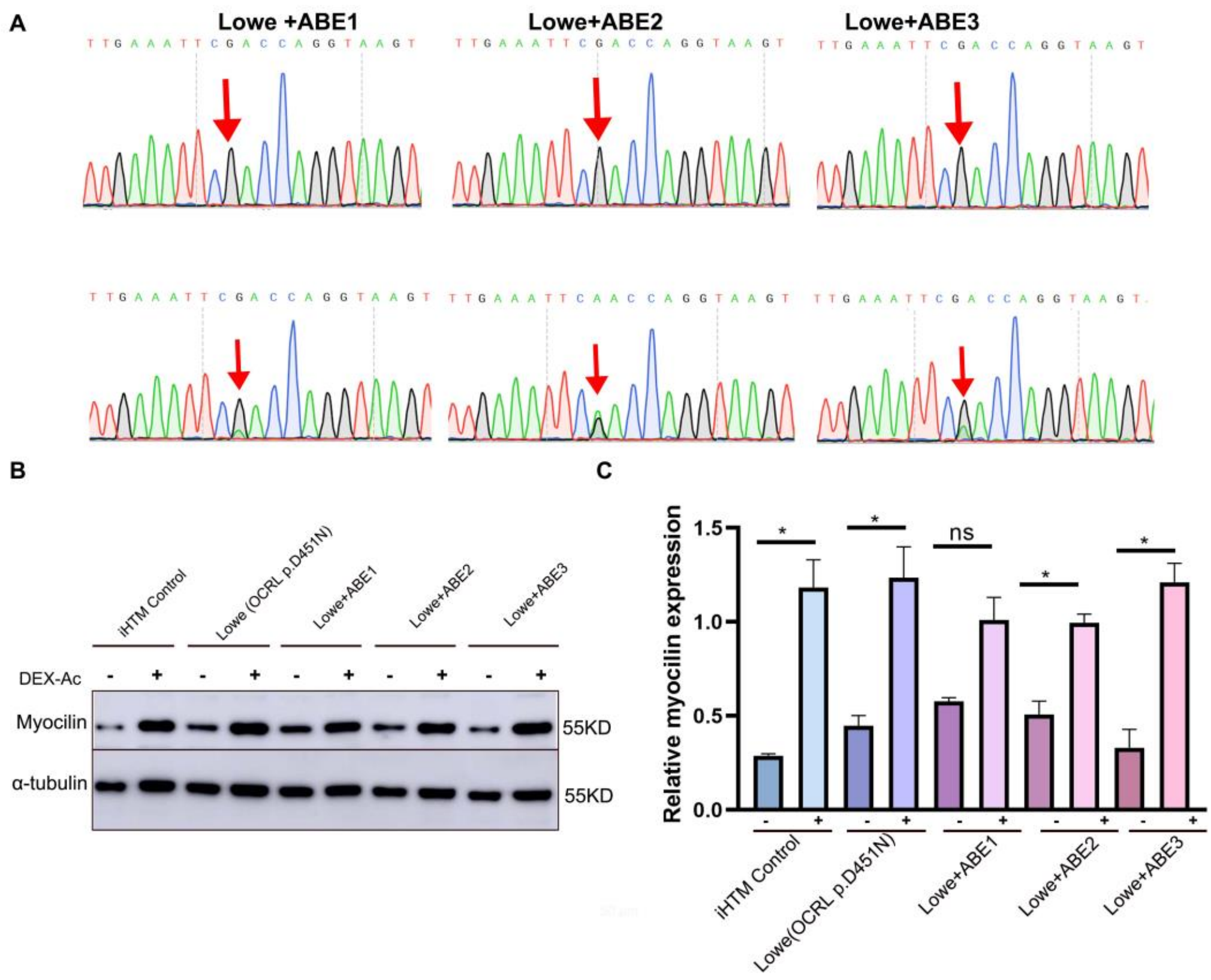

Supplementary Figure 5 **Base editing-mediated correction of *OCRL* mutation in Lowe patient iPSCs.** (A) Representative Sanger sequencing chromatograms of iPSCs clonal isolation results. Red arrows indicate nucleotides corrected by SpRY-ABE8e. (B) Representative Western blot analysis showing Myocilin protein levels in iHTM control cells, OCRL mutant cell line (OCRL p.D451N) and three OCRL mutation-corrected cell lines (Lowe+ABE1/2/3) following 14 days of incubation in the absence (-) or presence (+) of 100 nM DEX-Ac. α-tubulin was utilized as an internal loading control. (C) Quantitative assessment of relative Myocilin of the Western blot bands normalized to α-tubulin loading controls. Data represent (mean ± SEM n=2 independent experiments). Statistical significance between untreated and treated groups within each cell line was evaluated using a Student's t-test (* P<0.05; ns, non-significant).

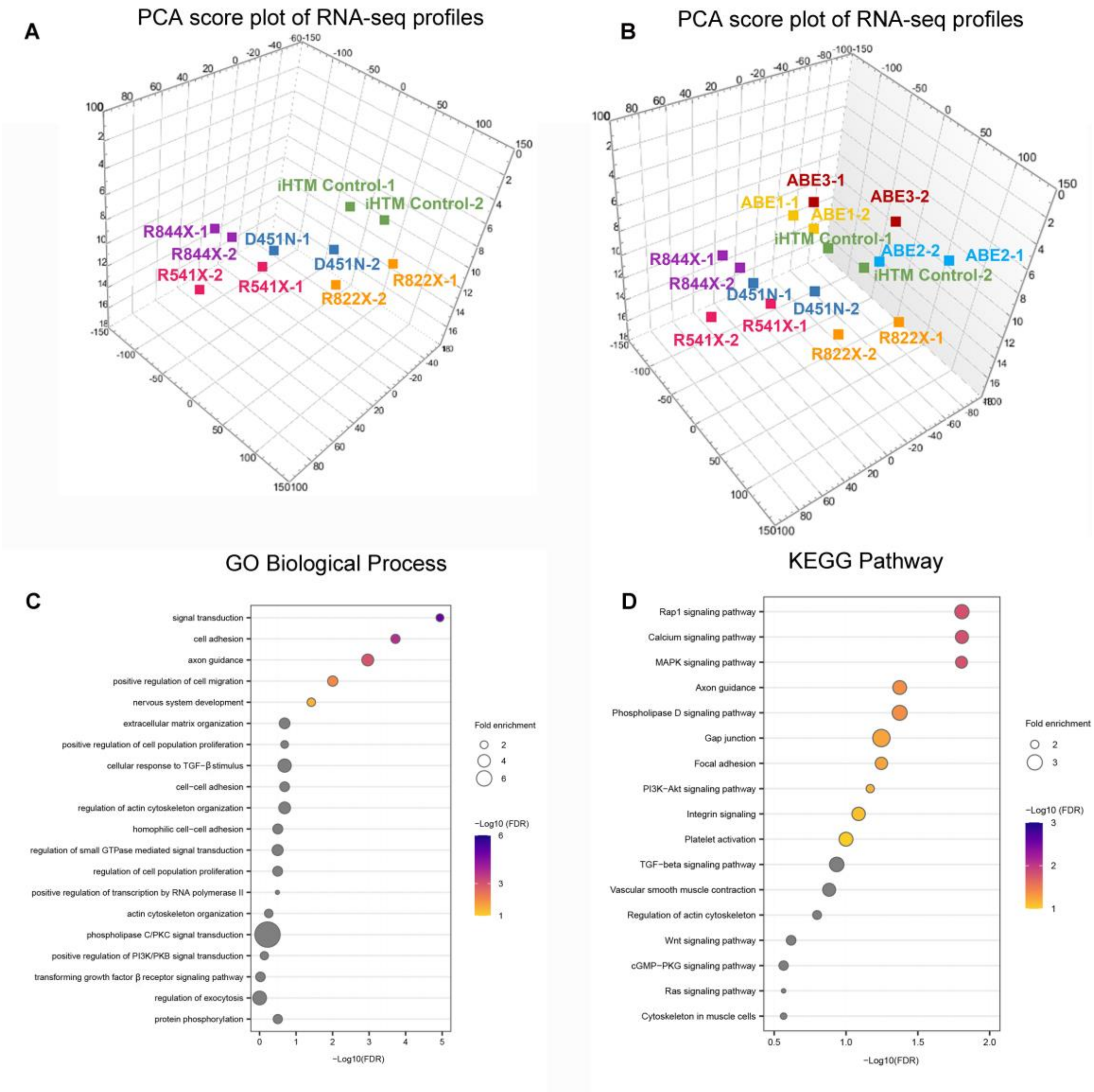

Supplementary Figure 6 **Transcriptomic profiling of control, Lowe syndrome, and gene-corrected iHTM cells.** (A) Principal component analysis of RNA-seq profiles from control and Lowe syndrome iHTM cells. PCA was performed in SIMCA using RNA-seq gene expression data. Each point represents one biological replicate, and colors indicate the corresponding cell line. PC1 and PC2 explained 48.0% and 20.6% of the total variance, respectively. (B) PCA of RNA-seq profiles from control iHTM cells, Lowe syndrome iHTM cells harboring *OCRL* variants (p.D451N, p.R541X, p.R822X, and p.R844X), and three independently mutation-corrected Lowe iHTM clones (ABE1-ABE3). Each point represents an individual RNA-seq sample. Samples are colored according to cell line or treatment group. (C) Gene Ontology (GO) Biological Process enrichment analysis of differentially expressed genes. Dot size represents fold enrichment, and color indicates statistical significance based on -$\log_{10}$(FDR). (D) Kyoto Encyclopedia of Genes and Genomes (KEGG) pathway enrichment analysis of differentially expressed genes. Dot size represents fold enrichment, and color indicates -$\log_{10}$(FDR).
